# Identification of functional butanol-tolerant genes from *Escherichia coli* mutants derived from error-prone PCR-based whole-genome shuffling

**DOI:** 10.1186/s13068-019-1405-z

**Published:** 2019-04-01

**Authors:** Xueting He, Tingli Xue, Yuanyuan Ma, Junyan Zhang, Zhiquan Wang, Jiefang Hong, Lanfeng Hui, Jianjun Qiao, Hao Song, Minhua Zhang

**Affiliations:** 1 Biomass Conversion Laboratory, R&D Center for Petrochemical Technology, Tianjin University, Tianjin, 300072 People’s Republic of China; 2 Department of Biochemical Engineering, School of Chemical Engineering and Technology, Tianjin University, Tianjin, 300072 People’s Republic of China; 3 Frontier Technology Research Institute, Tianjin University, Tianjin, 30072 People’s Republic of China; 4 Collaborative Innovation Centre of Chemical Science and Engineering (Tianjin), Tianjin, 300072 China; 5 Key Laboratory of Systems Bioengineering (Ministry of Education), SynBio Research Platform, Tianjin University, Tianjin, 300072 China; 6 Tianjin Key Laboratory of Pulp and Paper, Tianjin University of Science and Technology, Tianjin, 300457 China

**Keywords:** *Escherichia coli*, Butanol-tolerant mutants, Comparative functional genome, Rob, AcrB, Transcription regulator factor, Efflux pump, Quorum-sensing signal transporter

## Abstract

**Background:**

Butanol is an important biofuel and chemical. The development of butanol-tolerant strains and the identification of functional butanol-tolerant genes is essential for high-yield bio-butanol production due to the toxicity of butanol.

**Results:**

*Escherichia coli* BW25113 was subjected for the first time to error-prone PCR-based whole-genome shuffling. The resulting mutants BW1847 and BW1857 were found to tolerate 2% (v/v) butanol and short-chain alcohols, including ethanol, isobutanol, and 1-pentanol. The mutants exhibited good stability under butanol stress, indicating that they are potential host strains for the construction of butanol pathways. BW1847 had better butanol tolerance than BW1857 under 0–0.75% (v/v) butanol stress, but showed a lower tolerance than BW1857 under 1.25–2% (v/v) butanol stress. Genome resequencing and PCR confirmation revealed that BW1847 and BW1857 had nine and seven single nucleotide polymorphisms, respectively, and a common 14-kb deletion. Functional complementation experiments of the SNPs and deleted genes demonstrated that the mutations of *acrB* and *rob* gene and the deletion of *TqsA* increased the tolerance of the two mutants to butanol. Genome-wide site-specific mutated strains DT385 (*acrB* C_1198_T) and DT900 (*rob* AT_686–7_) also showed significant tolerance to butanol and had higher butanol efflux ability than the control, further demonstrating that their mutations yield an inactive protein that enhances butanol resistance characteristics.

**Conclusions:**

Stable *E. coli* mutants with enhanced short alcohols and high concentrations of butanol tolerance were obtained through a rapid and effective method. The key genes of butanol tolerance in the two mutants were identified by comparative functional genomic analysis.

**Electronic supplementary material:**

The online version of this article (10.1186/s13068-019-1405-z) contains supplementary material, which is available to authorized users.

## Background

Butanol is a potential superior alternative to ethanol as biofuel, with a higher energy density, lower hygroscopicity and volatility, and less corrosive. Butanol has also been used in a wide range of fields, including the food, plastics, and pharmaceutical industries [1]. Engineering a butanol biosynthetic pathway into *Escherichia coli* (*E. coli*) could produce *n*-butanol at the level of grams per liter and compete with the traditional industrial producer, genus *Clostridium* [1–3]. However, the poor butanol tolerance of bacteria leads to the inhibition of cell growth and a decrease in butanol yield, which has been a bottleneck in biobutanol production. Thus, the economic production of butanol relies on the improvement in butanol tolerance of the bacterial producers [4, 5].

To improve the robustness of chassis strains, rational metabolic engineering strategies have been used to generate butanol or other fuel-tolerant mutants of *E. coli* [4, 6]. Genes in relation to membrane function and ion transport system have been reported to respond to butanol stress, the tolerance to which could be improved by the overexpression of genes encoding the efflux pump [7–9], ion transport proteins entC and feoA [10, 11], and membrane-targeted metallothionein [12]. Heat shock protein (HSP) genes also respond to butanol stress; as such, their upregulation or overexpression could also lead to an increase in tolerance [13].

The mechanism of butanol tolerance, thus, involves multiple physiological processes mediated by a gene network. Butanol-tolerant *E. coli* strains are also obtained using random engineering strategies, such as global transcription machinery engineering (GTME) and evolution engineering, among others [14–17]. These mutants developed by random mutation and selection generally show a greater tolerance than those with functional changes in one gene, and could tolerate 1–2.0% (v/v) butanol [16, 18]. This may due to the fact that multiple mutated genes endow strains with a greater tolerance. When membrane-related functional genes are overexpressed in butanol-tolerant *E. coli* through evolution, the final engineered strain can grow under 2% butanol stress [18], indicating that a combinational strategy could effectively improve tolerance. If the key butanol-tolerant genes could be identified and clarified, these favorable mutations could be introduced to chassis strain, also referred to as inverse metabolic engineering [19]. Nevertheless, the genes of many mutants have not yet been functionally identified, which limits our understanding of the butanol tolerance mechanism as well as the potential to obtain butanol-tolerant characteristics.

It is still challenging to develop high butanol-tolerant strains using metabolic engineering or combinatorial approach because of the current limited understanding of the tolerance mechanisms; it is first necessary to establish a simple and rapid evolution strategy for the acquisition of resistant mutants, to thereafter identify the functional stress-tolerant genes and elucidate the mechanism of butanol tolerance. In this study, error-prone PCR (epPCR)-based whole-genome shuffling was used to improve the butanol tolerance of *E. coli* BW25113. As a result, two mutants tolerating 2% (v/v) butanol were successfully obtained. The mutation sites of the two strains were then identified by whole-genome resequencing, and comparative functional genomic analysis was performed to identify the key butanol-tolerant genes.

## Results

### Isolation and characterization of butanol-tolerant mutants by error-prone PCR-based whole-genome shuffling

**Fig. 1:**
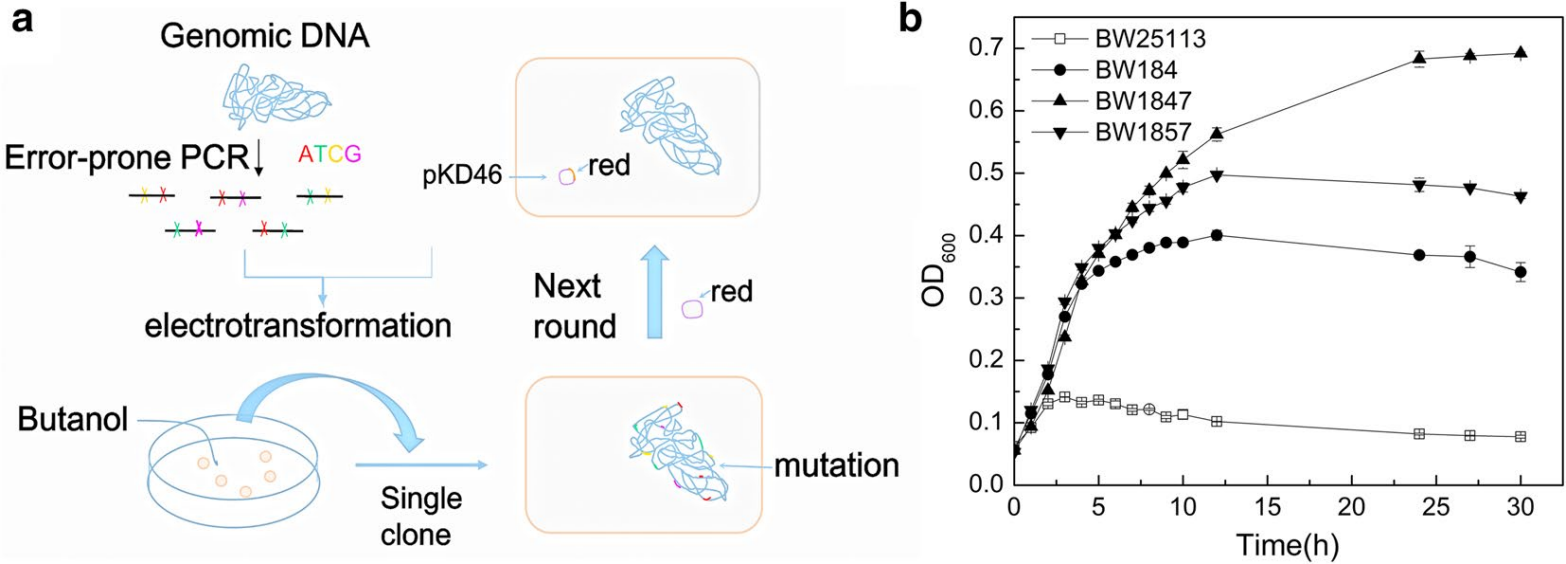
Screening of butanol-tolerant *E. coli* strains by epPCR-based genome shuffling. **a** Schematic of epPCR genome shuffling. **b** Growth of transformants under 0.95% (v/v) butanol stress

Error-prone PCR-based (epPCR) genome shuffling was used to screen butanol-tolerant strains. Using this method, epPCR products were integrated into the genome of host cells by electro-transformation (Fig. 1), [20, 21]. As such, BW25113 (pKD46) was used as an initial strain to improve recombinant efficiency via lambda-Red recombinase genes in a pKD46 plasmid. Strains exhibiting a butanol-tolerant phenotype were obtained by the first round of shuffling, where BW184 presented the best tolerance. The cell density of BW184 increased eightfold from the initial levels under 1.0% (v/v) (always expressed as v/v) butanol stress at 12 h, whereas the growth of BW25113 was completely inhibited. BW184 was, thus, used as a starting strain for next round of genome shuffling, and the pKD46 plasmid was transferred to BW184 for high-efficient recombination of PCR products to genome. The mutant strains BW1847 and BW1857 were obtained from the second round of shuffling, reaching a maximum OD_600_ of 0.69 and 0.50, respectively, which was 5.0- and 3.6-fold higher than that of BW25113, respectively. BW1847 was delayed 12 h to enter a stable phase (Fig. 1). The two strains, thus, showed more tolerance than both the initial strain and BW184, and their tolerance properties were further investigated.

### Tolerance of BW1847 and BW1857 to various short-chain alcohols

**Fig. 2:**
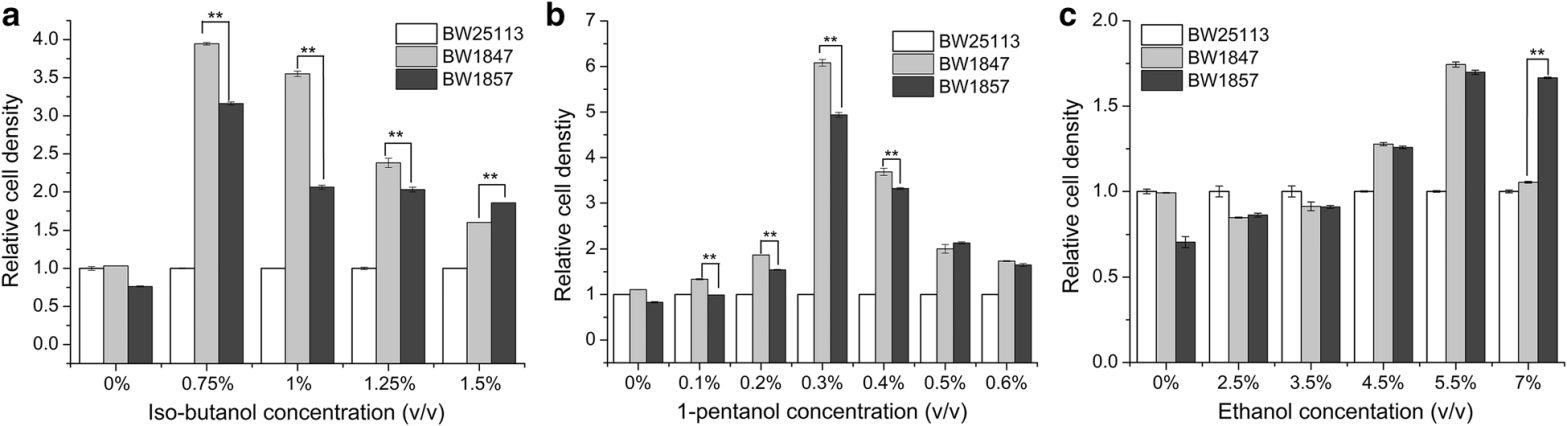
Relative cell densities of strains in the presence of various concentrations of short-chain alcohols. Strains were cultured under different concentrations of iso-butanol (**a**), ethanol (**b**) and 1-pentanol (**c**), and their OD_600_ was determined. The relative cell density is expressed as a ratio of the biomass value of mutants to that of parallel culture BW25113 under the same culture conditions

BW1847 and BW1857 had a higher tolerance to 0.75–1.5% (v/v) isobutanol, 0.1–0.6% 1-pentanol, and 4.5–7% ethanol (Fig. 2) compared to the control. BW1847 showed greater tolerance to isobutanol than BW1857 in a relatively low concentration (0.75–1.25%) of isobutanol stress. Nevertheless, it had a relative low cell density compared to BW1857 under 1.5% isobutanol and 7% ethanol stress, which also demonstrated that BW1857 had a better tolerance to high concentrations of isobutanol and ethanol. Interestingly, this trend was similar to that observed in subsequent butanol stress experiments. BW1847also had higher relative cell densities than BW1857 in the presence of 0.1–0.4% 1-pentanol, indicating that BW1847 had a greater tolerance to 1-pentanol. These results indicate that the mutant strains exhibited resistance to short-chain alcohols with different tolerance characteristics.

### Growth and stability under varying butanol stress

**Fig. 3:**
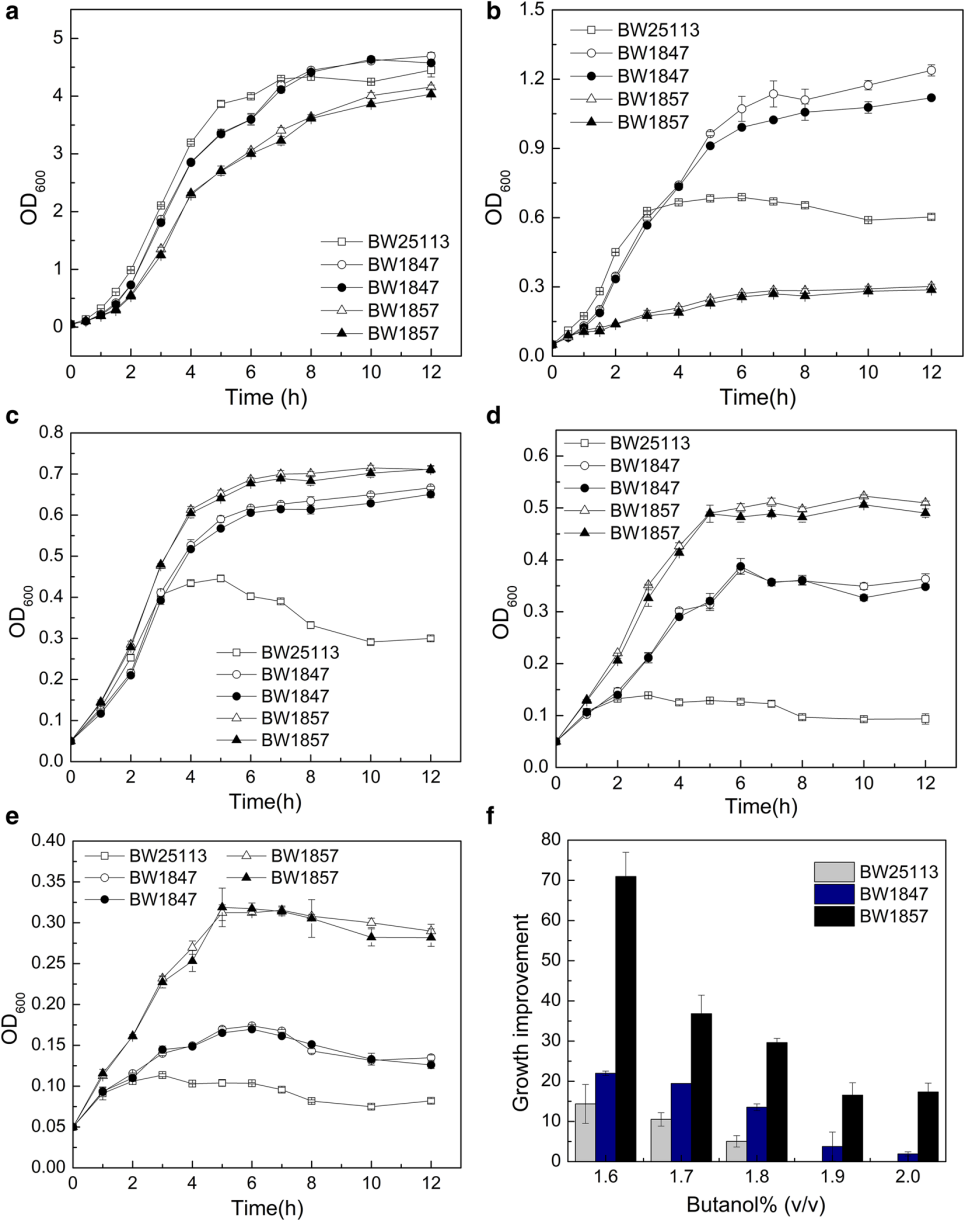
Evaluation of butanol tolerance stability and high-concentration butanol tolerance of BW1847 and BW1857. Strains were cultured in LB containing 0 (**a**), 0.75 (**b**), 1.0 (**c**), 1.25 (**d**), or 1.5% (**e**) (v/v) butanol. Solid and hollow symbols indicate the strains subjected or not, to 50 generations of subculture, respectively. Rectangles, circles, and triangles represent the initial strains BW25113, BW1847, and BW1857, respectively. **f** Growth of strains cultured in 1.6–2.0% butanol

BW1847 and BW1857 strains were serially subcultured to 50 generations in LB media without butanol for stability evaluation of butanol tolerance. Both subcultured and non-subcultured strains of 50 generations were synchronously cultured for growth detection. Strains subjected to 50 generations of subculture had similar growth curve profiles to those not subjected to subculture in the presence of 0–1.5% butanol, indicating that BW1847 and BW1857had good butanol-tolerance stability (Fig. 3a–e), making them promising candidates for further industrial application.

BW1857 showed slower growth and lower cell density than BW25113 and BW1847 both under 0.75% butanol stress conditions and without butanol, demonstrating that growth of BW1857 was inhibited (Fig. 3a, b). However, BW1857 had a higher cell density than BW1847 in the presence of 1–1.5% butanol, indicating that BW1857 exhibited a better tolerance in high butanol concentrations (Fig. 3c–e). The cell densities of BW1847 and BW1857 were 68% and 204% higher, respectively, than that of the control BW25113 at 1.5% butanol, demonstrating their tolerance to high concentrations of butanol (Fig. 3e). BW1857 showed an inhibited and enhanced growth under low and high concentrations of butanol, respectively, implying that it contains multiple gene mutations, some of which may lead to improved butanol tolerance, with others resulting in growth inhibition.

Growth under high concentrations of butanol was evaluated (Fig. 3f). BW1847 and BW1857 showed 1.5- and 2.7-fold, and 3.5- and 5.9-fold increased growth compared to the wild-type in the presence of 1.6 and 1.8% butanol, respectively. The cell growth of BW1847 and BW1857 was 1.9–3.7% and 16.5–17.3%, respectively, of the control strain under high butanol stress (1.9–2.0%). These data demonstrate that the butanol tolerance of the two mutants was significantly higher than that of BW25113, and they could tolerate 2% butanol. BW1857 showed a relatively higher tolerance than BW1847 in concentrations of butanol above 1.25% (Fig. 3c–f).

### Genome-wide identification of mutations by comparative genomics

The mutations in the genomes of BW1847 and BW1857 were identified by resequencing using the genome sequence of the starting strain, BW25113 (NZ_CP009273.1), as a reference control. PCR amplifications of the mutated (Table 1) and deleted genes were then performed to confirm the changes in the two mutants (Additional file 1: Additional data). Both BW1847 and BW1857 had a deletion fragment of 14 kb containing seventeen genes (Additional file 1: Additional data, Figure S1; Table S1). BW1847 and BW1857 had a total of nine and seven mutations, respectively. They had five common single nucleotide polymorphisms (SNPs) (Table 1), and an extra four and two mutations in BW1847 and BW1857, respectively. Of the 11 mutations, seven were missense mutations and four were nonsense mutations (Table 1).

**Table 1:** Mutations identified in the BW1847 and BW1857 strains

Strains	Gene/LC	Function	Mutation^a^	Gene position/length	Genome position	Effect^b^
BW1847 and BW1857	*cdsA/*RS00875	Phosphatidate cytidylyltransferase	G/T	751/858	192,914	G (251) C
	*pgsA/*RS10005	CDP-diacylglycerol-glycerol-3-phosphate 3-phosphatidyltransferase	T/G	446/594	1,985,853	V (149) G
	*yheQ*/RS11070	Hypothetical protein	T/G	1843/1845	2,204,421	E (615)^c^
	*hycD*/RS14165	Formate hydrogenlyase subunit	C/A	899/1572	2,839,873	A (300) E
	*aslB*/RS19735	Anaerobic sulfatase maturase AslB	T/A	1223/1538	3,977,540	V (408) E
	*RS08320*–*RS08395*				1,658,639–1,672,922	14-kb deletion
BW1847	*acrB*/RS02385	Multidrug efflux RND transporter permease subunit AcrB	C/T	1198/3792	478,662	L (400) F
	*spoT*/RS18950	Bifunctional (p)ppGpp synthetase II/guanosine-3′,5′-bis pyrophosphate 3′-pyrophosphohydrolase	G/T	1882/2109	3,817,641	E (628)^c^
	*nusG*/RS20660	nusG transcription termination/antitermination	G/T	436/546	4,168,106	G (146) C
	*rob*/RS22900	Right oriC-binding transcriptional activator, AraC family	AT/	1379	4,624,441 (2)	Deletion^c^
BW1857	*rplB*/RS17195	50S ribosomal protein L2	T/A	491/822	3,444,233	I (164) N
	*infB*/RS16425	Translation initiation factor IF-2		331–555/2673	3,308,845–3,309,069	225 bp deletion^c^

*LC* locus number

^a^The nucleotide on the left of the backslash was deleted in the corresponding gene, and that on the right of the backslash was inserted in the corresponding gene

^b^Amino acid position is in parentheses, amino acid residue on the left and right side of parentheses denotes the original and that substituted in the corresponding protein, respectively

^c^Termination codon

### Functional identification of mutated genes in BW1847 and BW1857

**Fig. 4:**
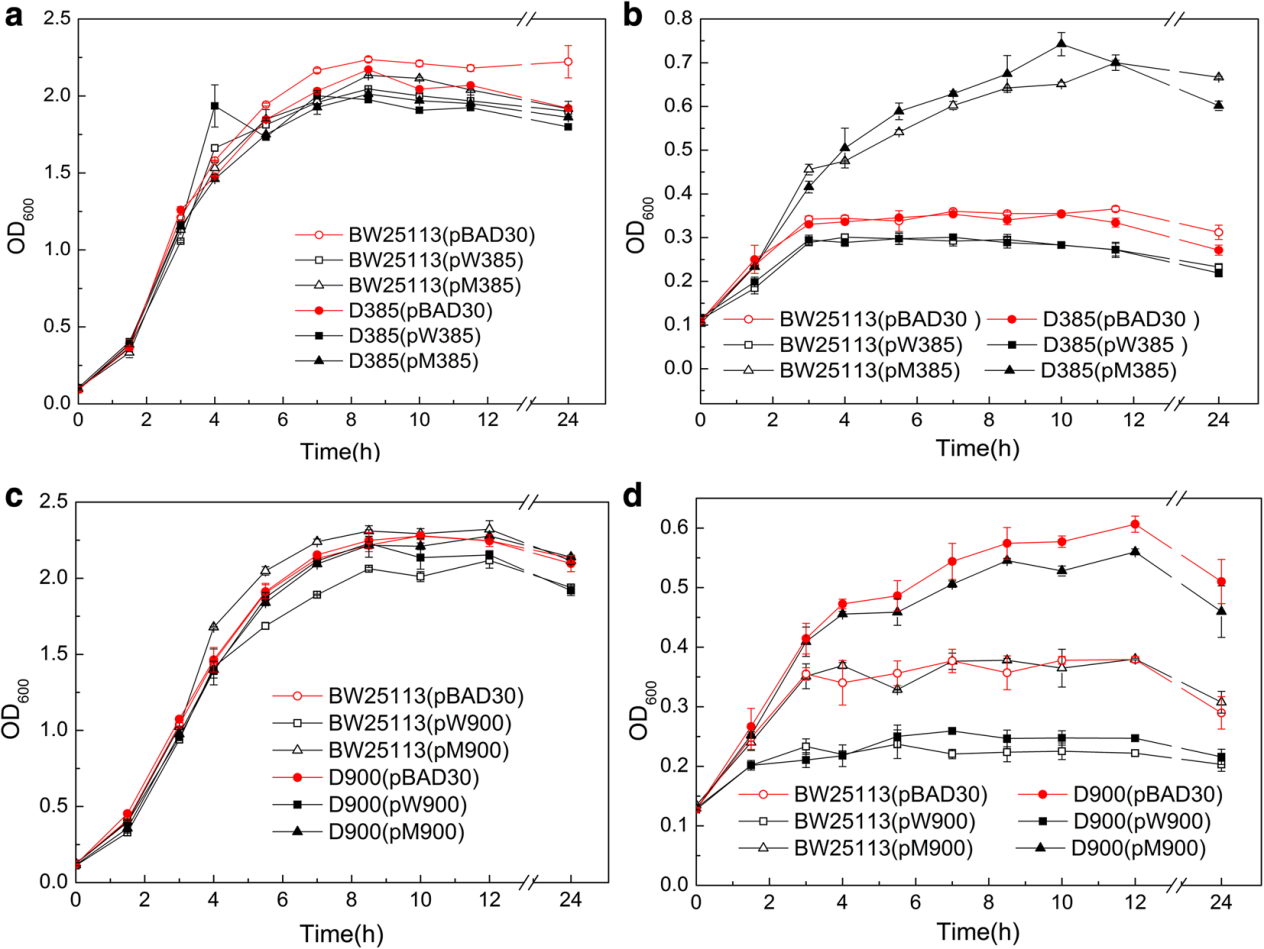
Growth evaluation of *acrB* and *rob* overexpression strains. Serial *acrB* (**a**) and *rob* (**b**) gene overexpression strains were cultured in LB media with (**b**, **d**) and without butanol (**a**, **c**). The initial concentration of inoculation was controlled at OD_600_ = 0.2, and 0.02% (w/v) of L-arabinose was added to the media to induce the expression of the target gene

Functional complementation experiments of each mutated gene (Tables 1, 2) were performed to examine their involvement in *n*-butanol tolerance (Additional file 1: Tables S1–S6, Additional data). When the functional complementary strains of the *acrB* gene (RS02385) were cultured in media without butanol, they all showed similar growth trends, indicating that the deletion and overexpression of *acrB* gene did not affect growth (Fig. 4a). The maximum cell density of the mutated *acrB* (1198C > T) overexpression strains, BW25113 (pM385) and D385 (pM385), was 1.9- to 2.1-fold higher than the corresponding control strains, BW25113 (pBAD30) and D385 (pBAD30), under 0.7% butanol stress. Nevertheless, the growth of *acrB* overexpression strains, BW25113 (pW385) and D385 (pW385), decreased 13.5–18.9% compared to that of the controls, BW25113 (pBAD30) and D385 (pBAD30)(Fig. 4b), indicating that the mutation (1198C > T) of *acrB* causes an increase in the butanol tolerance of BW1847. The cell density of BW25113 (pM385) was significantly increased compared to that of BW25113 (pBAD30), indicating that the 1198C > T mutation must be dominant since it significantly improves the tolerance even when wild-type acrB is present.

**Table 2:** Strains used in functional complementary study

Strains	Description	Sources
*E. coli* Top10F′	Host for plasmid construction	Lab stock
*E. coli* BW25113	Host for plasmid expression, F^−^ Δ(*araD*-*araB*)567 Δ*lacZ4787(::rrnB*-*3)*λ^−^*rph*-*1* Δ*(rhaD*-*rhaB)568 hsdR514*	Lab stock
BW25113 (pBAD30)	pBAD30 is a arabinose-inducible expression vector, with ori 15A replicon, Ap^R^	This study
BW25113 (pW/M005)	BW25113 (pBAD30 carries wild/mutated *pgsA*)	This study
BW25113 (pW/M070)	BW25113 (pBAD30 carries wild/mutated *yehQ*)	This study
BW25113 (pW/M165)	BW25113 (pBAD30 carries wild/mutated *hycD*)	This study
BW25113 (pW/M735)	BW25113 (pBAD30 carries wild/mutated *aslB*)	This study
BW25113 (pW/M875)	BW25113 (pBAD30 carries wild/mutated *cdsA*)	This study
BW25113 (pW/M385)	BW25113 (pBAD30 carries wild/mutated *acrB*)	This study
BW25113 (pW/M660)	BW25113 (pBAD30 carries wild/mutated *nusG*)	This study
BW25113 (pW/M900)	BW25113 (pBAD30 carries wild/mutated *rob*)	This study
BW25113 (pW/M950)	BW25113 (pBAD30 carries wild/mutated *spoT*)	This study
BW25113 (pW/M425)	BW25113 (pBAD30 carries wild/mutated *infB*)	This study
BW25113 (pW/M195)	BW25113 (pBAD30 carries wild/mutated *rplB*)	This study
D005 (W/M005)	BW25113 Δ*pgsA::*cm^r^ (pBAD30 carries wild/mutated *pgsA*)	This study
D070 (pW/M070)	BW25113 Δ*yehQ::*cm^r^ (pBAD30 carries wild/mutated *yehQ*)	This study
D165 (pW/M165)	BW25113 Δ*hycD*::cm^r^ (pBAD30 carries wild/mutated *hycD*)	This study
D735 (pW/M735)	BW25113 Δ*aslB*::cm^r^ (pBAD30 carries wild/mutated *aslB*)	This study
D875 (pW/M875)	BW25113, Δ*cdsA::*cm^r^ (pBAD30 carries wild/mutated *cdsA*)	This study
D385 (pW/M385)	BW25113, Δ*acrB::*cm^r^ (pBAD30 carries wild/mutated *acrB*)	This study
D660 (pW/M660)	BW25113, Δ*nusG::*cm^r^ (pBAD30 carries wild/mutated *nusG*)	This study
D900 (pW/M900)	BW25113, Δ*rob*::cm^r^ (pBAD30 carries wild/mutated *rob*)	This study
D950 (pW/M950)	BW25113, Δ*spoT::*cm^r^ (pBAD30 carries wild/mutated *spoT*)	This study
D425 (pW/M425)	BW25113, Δ*infB*::cm^r^ (pBAD30 carries wild/mutated *infB*)	This study

The deletion and overexpression of the *rob* gene (RS22900) also had no influence on growth (Fig. 4c). The *rob* deletion mutant D900 (pBAD30) showed 1.6-fold higher maximum cell density than BW25113 (pBAD30). Nevertheless, the overexpression of the *rob* gene resulted in a 37.5–57.0% decrease of growth under 0.7% butanol stress, indicating that the deficiency of *rob* led to an enhanced butanol tolerance. The complementation strain D900 (pM900) showed an inhibited growth similar to that of D900 (pBAD30) under butanol stress (Fig. 4d), indicating that the overexpression of the truncated *rob* gene was unable to restore the function of *rob*. This also demonstrates that the deletion of the AT_686–7_ base in the *rob* gene in BW1847 causes an inactivation of Rob function, which improves the butanol tolerance of BW1847. Another 9 mutated genes, including *yhcD* (RS14165) and *nusG* (RS20660), were identified but had no distinct influence on growth or butanol tolerance.

### Functional identification of deleted genes

The deleted 14-kb fragment contained 17 genes, in which the *pntA* (RS08390) and *pntB* (RS08385) belonged to one operon and encoded the alpha and beta subunits of pyridine nucleotide transhydrogenase. The deletion mutant of each gene and mutant D123 with simultaneous deletion of the 17 genes (Additional file 1: Table S1) were constructed to test the butanol-tolerant function of these genes (Additional file 1: Table S5, Additional data). The large fragment deletion caused a slight growth inhibition but resulted in a slight improvement of butanol tolerance under 1–1.5% (v/v) butanol stress (Additional file 1: Figure S2).

The deletion of each gene among the 17 genes did not affect growth. Gene *TqsA* (RS08380) deletion improved butanol tolerance performance of strains under high concentrations of butanol (above 1%) stress, but not under relatively low-concentration (0.5–0.75%) butanol stress (Table 3). The growth improvement of D380 was 34.6%, 67.8% and 82.8% higher than that of BW25113 under 1%, 1.25% and 1.5% (v/v) butanol stress, respectively. The growth of D380 and BW25113 was both inhibited under 1.75% butanol stress, but the biomass of D380 was 136% higher than that of the control (Table 3). The overexpression of *﻿TqsA* decreased butanol tolerance (Additional file 1: Figure S3). These data demonstrate that D380 has a significantly enhanced tolerance to increased butanol concentration of 1–1.5% (Table 3; Additional file 1: Figure S3). The deletion of *dgsA* (RS08340) and *mdtJ* (RS08375) led to a slight decrease in butanol tolerance (Additional file 1: Figure S4B, C; Table S1). Nevertheless, the overexpression of *dgsA* and *mdtJ* did not restore the tolerance level, which may be either due to plasmid and inducer interference or because neither gene has a significant tolerance function. The deletion of another 13 genes did not affect growth or butanol tolerance. Therefore, the deletion of *TqsA* plays an important role in tolerance under relatively high concentrations of butanol stress.

**Table 3:** Growth improvement of the *TqsA* deletion mutant D380

Butanol % (V/V)	BW25113	D380
0.75	156.6±4.1	122.1±1.5
1	52.8±3.1	70.5± 2.0
1.25	18.5±0.6	31.0±0.7
1.5	6.0 ± 0.6	10.9±0.8
1.75	1.4±1.43	3.3 ± 1.0

D380 was cultured in LB containing 0.75–1.75% butanol, and its growth improvement was calculated according to formula (1) as described in “Methods” section

Multiple gene-deleted strains, D1 (with deletion of genes RS08320–RS08335), D2 (with deletion of genes RS08340–RS08375), D3 (with deletion of genes RS08380–RS08395), D12, D13, and D23, were constructed to investigate the effect of combined deletion on growth and tolerance. The growth and tolerance of the D2, D12, D23, and D13 strains were lower than that of the other strains, indicating that the deletion of RS08340–RS08375 leads to a decrease in growth (Additional file 1: Figure S5). The deleted fragment contained *dgsA* and *mdtJ*, further indicating that the simultaneous deletion of these two genes causes a slight inhibition of growth and butanol resistance. Therefore, the deletion of *TqsA* is a key factor for improving the butanol tolerance characteristics of D123, BW1847, and BW1857 grown under 1–1.5% butanol stress.

### Butanol tolerance function of *rob* and *acrB* genes

**Fig. 5:**
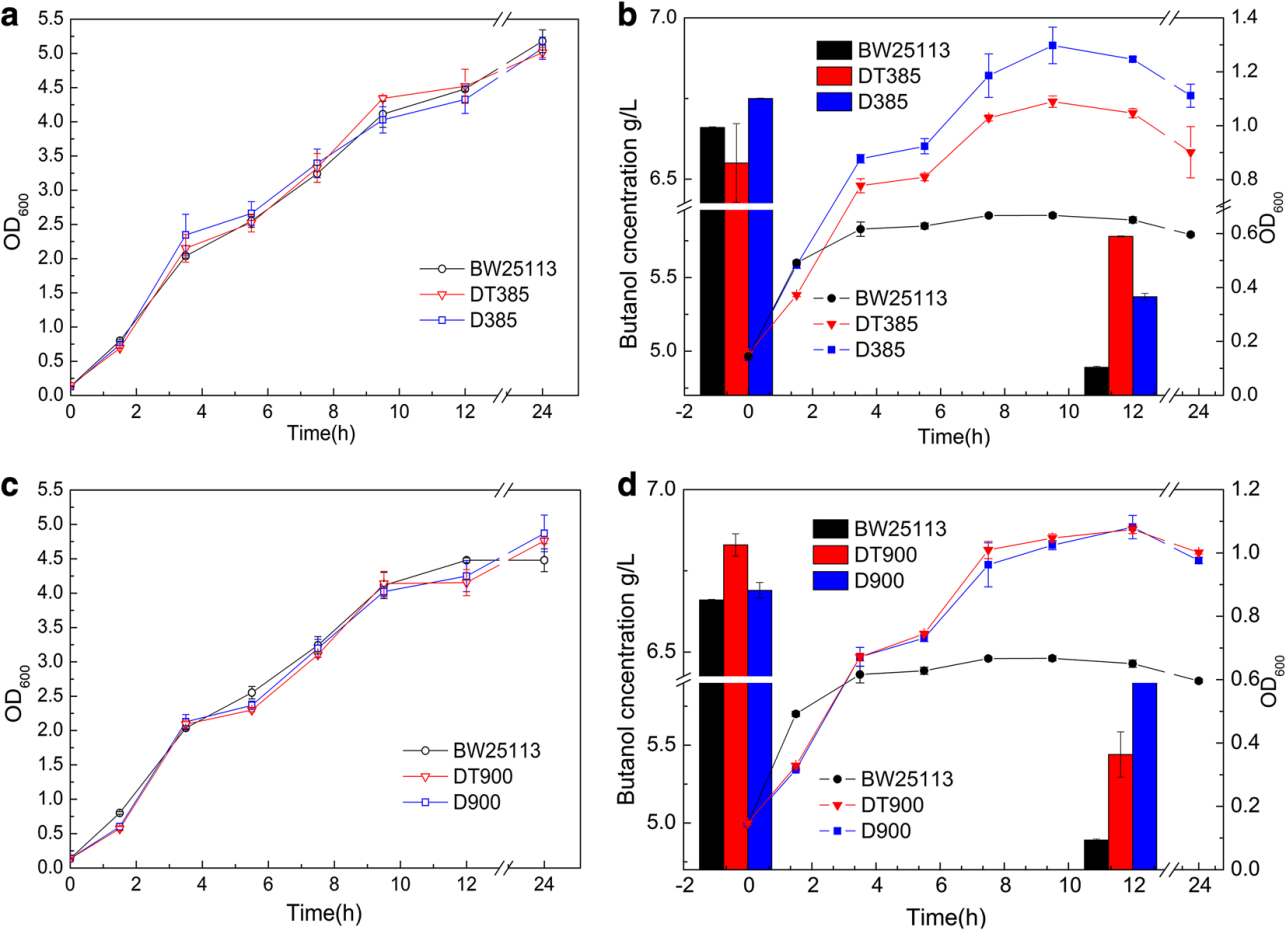
Growth curve and butanol efflux of site-specific mutants. The mutant DT385 with mutated *acrB*_C1198T_ and DT900 with a truncated *rob*_AT686–7_ gene were obtained by genome-wide site-specific methods. The two strains and their gene-deleted mutants D385 and D900 were cultured in LB with (**b**, **d**) or without (**a**, **c**) 0.75% butanol. The left and right axes indicate the butanol concentration in media and the cell density (OD_600_), respectively

Two genome-wide site-specific mutants, DT385 (mutation of C_1198_T in *acrB* gene) and DT900 (AT_686–7_ deletion in *rob* gene), were constructed to study the effect of *acrB* and *rob* gene mutations on butanol tolerance and to eliminate the effect of plasmid and inducer arabinose on growth during tolerance evaluation. AcrB (formerly AcrE), a multidrug efflux RND transporter permease subunit, interacts with membrane fusion protein AcrA and outer-membrane protein TolC to form drug-efflux systems, which can efflux the toxic compounds, such as geraniol, limonene, and pinene, but not butanol [6, 9, 22]. The growth curves of DT385 and the control strain grown in standard LB exhibited nearly identical trends, showing that the C_1198_T mutation of *acrB* (Table 1) had no influence on cell growth (Fig. 5a). However, the maximum cell densities of D385 and DT385 were 1.9- and 1.6-fold higher than that of BW25113, respectively, when cultured with 0.75% (v/v) butanol (Fig. 5b). The higher butanol concentration (5.37–5.78 g/L) was found in the media of the two mutants compared to that in the control (4.89 g/L). Moreover, the relative butanol content in one cell from D385 and DT385 was 0.60 and 0.54 g/L/OD_600_, respectively, which was lower than that of the control (2.72 g/L/OD_600_), indicating that the two strains had a higher butanol efflux ability than the control. These data demonstrate that both the deletion and point mutation of the *acrB* gene improved butanol tolerance as a result of the pumping of butanol out of the cells.

**Fig. 6:**
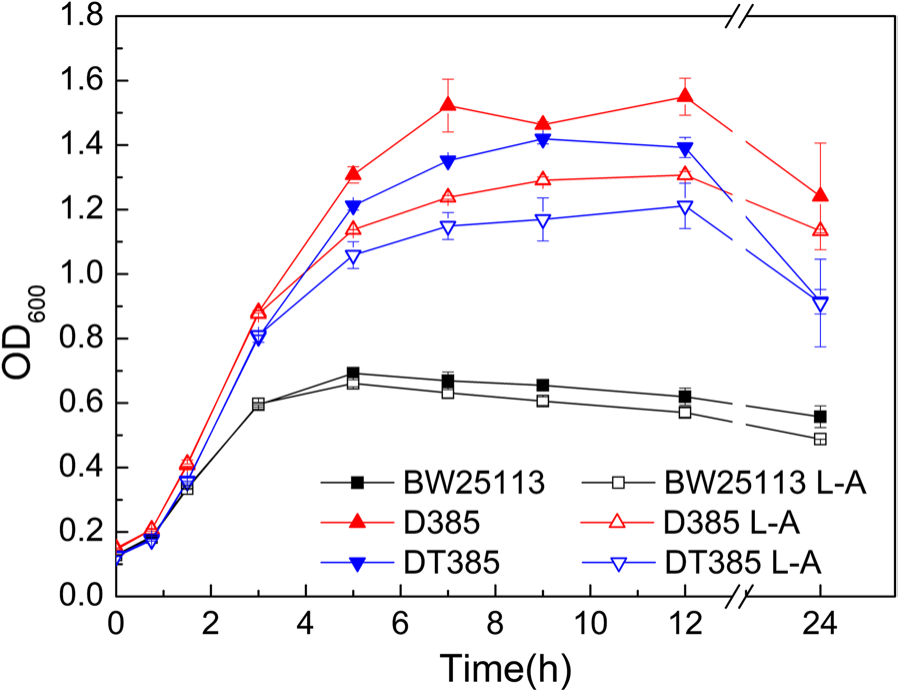
Growth curve of mutant D385 and DT385 grown under butanol stress with and without L-arabinose. Mutants and the control BW25113 were cultured in LB medium with 0.75% butanol. Solid and hollow symbols indicate the cell densities of the strains grown in media with or without arabinose, respectively

Interestingly, the functional complementation experiments showed that *acrB* deletion did not improve the butanol tolerance (Fig. 4). Arabinose was speculated to inhibit growth, such that D385 and DT385 were subsequently cultured in butanol media with and without L-arabinose for growth detection. Their maximum cell densities were lower (17.5–18.7%) with L-arabinose than without it (Fig. 6), demonstrating growth inhibition by the addition of L-arabinose. Nevertheless, the deletion strain D385 still showed tolerance characteristics (Fig. 6). The presence of pBAD30 serial plasmids in D385 strain results in that the tolerance of D385 is not displayed. The growth of D385 harboring the plasmids was probably negatively affected by the combined effect of the arabinose, butanol, and metabolic burden of plasmids, which may cause an absence of tolerance.

The *rob* gene encodes OriC-binding transcriptional activator, and AT_686–7_ deletion of *rob* in genome did not affect growth (Fig. 5c) but caused a significant increase in growth under 0.75% (v/v) butanol stress, indicating that deficiency of AT_686–7_ in *rob* can improve butanol tolerance(Fig. 5d). The *rob*-deleted strain D900 also showed higher tolerance, demonstrating that the truncated *rob* leads to its inactivation, causing an enhanced butanol tolerance. Moreover, the butanol efflux ability of the two mutants was also significantly improved (Fig. 5d). The inactivation of Rob could change the gene expression profile and corresponding physiological/biochemical processes under butanol stress. Some of the response genes regulated by Rob may function in the efflux of potentially toxic molecules to the outside of the cells.

## Discussion

Bacterial genomes can be conveniently and rapidly modified using epPCR whole-genome shuffling with a highly efficient recombination system. The recombination efficiency of *E. coli* recombinase *rec*A is very low [23]. Therefore, BW25113 (pKD46) containing a high-efficient recombinase system was chosen as the host. This resulted in two mutants, BW1847 and BW1857, which tolerated 2% butanol stress. Even though the growth of BW25113 decreased by 50% under 0.8% butanol stress [24], the two mutants in the study had a fourfold higher maximum cell density than BW25113 under 0.75% butanol stress, indicating a great improvement in their butanol tolerance. Until now, most *E. coli* strains with single gene modifications could only tolerate 0.75–1.5% butanol stress [7, 13]. However, the two mutants obtained in this study showed better tolerance than most reported strains. A few mutants tolerating 2% butanol have been previously obtained via combined strategies or multi-gene mutations [16, 18], by which the function of multiple genes involved in various physiology events is changed to respond to stress. Even though multiple mutations can easily change physiological and biochemical functions, thereby yielding a better tolerance, they can lead to a decrease in tolerance function. Consequently, the identification of key resistant genes is necessary.

Many butanol-tolerant strains are also reported to show relevant tolerance to other short-chain alcohols, such as ethanol, isobutanol, 1-pentanol, 1,2,4-butanetriol, and *n*-propanol [25, 26]. These short-chain alcohols possess similar molecular structures and exhibit toxicity. The fatty acid composition, structure, and fluidity of the cell membrane are similarly changed in response to stress by these short-chain alcohols. Therefore, the tolerance mechanism towards these alcohols is similar [27, 28].

BW1847 and BW1857 have 5 common SNPs, which also should be the mutations from BW184. However, functional complementation experiments carried out on each of these five genes did not confirm their butanol tolerance. As such, the tolerance of BW184 to butanol stress may result from the functional accumulation of all five mutated genes. The maximum cell density of BW1857 was lower than that of BW25113 and BW1847, and BW1857and BW1847 contained two different mutations, *infB*_deletion331–555_ and *rplB*_*T491A*_. Nevertheless, the functional complementation experiments of the two mutated genes did not elucidate growth inhibition. Moreover, the deletion mutant and the point mutant strain of *rplB* could not be successfully obtained due to the deletion being lethal, neither is it easily edited by Cas9-sgRNA. However, the deletion of a 14-kb fragment in both strains leads to slight growth inhibition, suggesting that the combined actions of the two SNPs and the deleted genes cause growth inhibition of BW1857.

Interestingly, a 14-kb deletion was observed in both mutants, but large DNA fragment deletion was not reported to be present in other mutants obtained by epPCR-based genome shuffling [21, 29], which is because only a few mutants, obtained using this technology, are analyzed by genome resequencing [20]. Consequently, an attempt was made to demonstrate the origin of the 14-kb deletion by experimentation and inference. If the deletion could be obtained by the lambda-Red recombination system of epPCR-based shuffling, then the deletion could have resulted from crossover recombination of a certain random epPCR product with coincident left and right homologous arm sequences of the target deletion fragment. The lambda-Red recombination system (pKD46), used in the shuffling, was used to knockout the target region, and twenty-nine transformants with 14-kb deletions were obtained (Additional file 1: Additional data, Figure S9, Table S11), indicating the possibility of deriving targeted deletions using this technology. Besides, final mutation rates of the two mutants were 1000-fold higher than the spontaneous mutation rates (Additional file 1: Additional data). Therefore, the 14-kb deletion was more likely due to homologous recombination of random epPCR products via lambda-Red recombinase used in the epPCR shuffling technology, than from spontaneous mutations during the recovery and screening phases after electroporation.

Three important tolerance genes were identified in this study: mutant *acrB*, mutant *rob*, and the deletion of *TqsA,* all increased butanol tolerance. Mutated AcrB_I466F_ is reported to pump butanol out of the cell, leading to an improvement in butanol tolerance [9]. AcrB_L400F_ in this study is also found to exhibit butanol-efflux ability, and the mutated I466F and L400F are both located on the transmembrane domain, and would change the spatial structure. AcrB forms a trimer composed of loose (L), tight (T), and open (O) conformational states. Toxic compounds were bound to the L monomer and then transported to the T monomer, before finally being excreted out of cells by the O monomer [22] (Additional file 1: Figure S6 A–C). The mutation in AcrB, thus, allowed for the binding and transportation of butanol, resulting in improved efflux function. Interestingly, *acrB* deficiency was reported to yield an improvement in butanol tolerance for the first time. This deletion resulted in an open funnel-shaped compound formed by AcrA and TolC in the periplasmic space (Additional file 1: Figure S6D), such that butanol was freely and directly transported out of cells by the uncompleted efflux pump system.

The transcription factor Rob responds to many stimuli, including antibiotics, organic solvents, and oxidative stressors, and its function in resistance to the stress is shown by overexpression of Rob through a plasmid [32–34]; while, the *rob*-deleted strains exhibit a decreased tolerance to the stresses [33, 34]. Nevertheless, the inactivation of Rob in this study enhanced the butanol tolerance (Figs. 4, 5). The Rob-inactive strain would have a different expression profile than the wild-type under butanol stress. The response genes, and thus the physiological roles regulated by Rob would be interesting and complex, and should be investigated to provide insights into the mechanism of butanol tolerance in future studies.

The deletion of *TqsA*, encoding an AI-2 transporter, was found to improve the butanol tolerance. Transmembrane protein TqsA transports quorum-sensing molecule AI-2 out of the cells, and its deletion increases the concentration of intracellular AI-2, which enhances the motility of cells, allowing cells to escape from toxic compounds [35, 36], and improves the biofilm thickness by up-regulating the expression of lipopolysaccharide (LPS)-synthesis-related genes [37, 38]. Therefore, the deletion of *TqsA* may lead to the improvement of intracellular AI-2, which subsequently increases cell motility and biofilm thickness, resulting in enhanced butanol tolerance.

Both the mutation and the deletion strain of *acrB* and *rob* improved growth by about 1.7- to 2.0-fold, compared to the control BW25113 under 0.75% butanol stress (Additional file 1: Table S10). The deletion of *TqsA* led to an improved tolerance (1.7-fold) in high concentration butanol media (1–1.25%). These three genes result in features for butanol tolerance, which could provide target genes for the engineering of a butanol-tolerant strain. The co-expression of these three genes is speculated to be able to result in an accumulated improvement of tolerance, which should be interesting and would be investigated further in future studies.

Among the 17 deleted and 11 mutated genes from the two mutants, only three genes were identified to exhibit significant butanol tolerance in this study. Correspondingly, among 16 mutated genes in mutant E72, only three mutations were beneficial [39]. These results demonstrate that only a few of multiple mutations may be valuable for functional improvement. Some mutations may produce an unobvious objective function or cause decreased target function. Therefore, improved characteristics in a mutant with random mutations may result from the combined actions of several key mutated genes. Consequently, the identification of key functional genes is important for revealing a corresponding mechanism and providing genetic resources for the construction of optimal chassis strains using rational metabolic engineering strategies.

## Conclusions

Two stable and high-concentration butanol-tolerant *E. coli* mutants were obtained by epPCR-based genome shuffling, a rapid and effective method. The two mutants were found to also tolerate short alcohols. Their mutation genes were revealed through genome resequencing. A comparative functional genomic study identified the key genes involved in butanol tolerance in both mutants, including *rob*, encoding transcript regulator factor Rob, *acrB*, encoding efflux pump transporter, and *tqsA*, encoding a quorum-sensing molecule transporter. The mutation or deletion of these three genes resulted in an improvement in butanol tolerance. Interestingly, the present data found for the first time that the deletion of *acrB* and *rob* yielded a significantly increased butanol-efflux ability, and the deletion of *tqsA* caused slight growth inhibition and high-concentration butanol-tolerance characteristics. These surprising and novel gene functions could provide a new point of view for the investigation of tolerance mechanism. This study, thus, provides valuable information for the construction of butanol-resistant strains and insights into the mechanism of butanol tolerance.

## Methods

### Strains and culture conditions

*E. coli* BW25113 (pKD46) was used as a starting strain. Strain BW1847 was deposited in the China Center for Type Culture Collection (CCTCC; Accession NO. M2015365). All *E. coli* strains were cultured in Luria–Bertani (LB) medium (5 g/L yeast extract, 10 g/L Casein tryptone, and 10 g/L NaCl) at 30 °C or 37 °C with 180–200 rpm shaking for growth.

### Error-prone PCR-based genome shuffling of *E. coli*

Genomic DNA of *E. coli* BW25113 was extracted from 2 mL of overnight cell culture and then used for the epPCR templates. EpPCR was conducted according to the protocols developed by Ye et al. [21] with some modifications [20] (Fig. 1a). The amplified PCR products were then concentrated 5- to 10-fold by ethanol-precipitated for electro-transformation (Additional file 1: Additional data).

*Escherichia coli* BW25113 (pKD46) were cultured in LB media with 100 μg/mL ampicillin at 30 °C until optical density at 600 nm (OD_600_) reached 0.15–0.2, and then L-arabinose was added to the media at a final concentration of 20 mM to induce expression of lambda-Red recombinase. When the cultures reached an OD_600_ of 0.45–0.5, electro-competent cells were prepared. Competent cells (40 μL) were mixed with 1–5 μL of ethanol-precipitated PCR products, and the mixture was incubated in ice bath for 5 min and then transferred to a pre-chilled 1-mm-gap electroporation cuvette (Eppendorf, Germany). The cells were electroporated with a pulse of 1800 v at 5 ms using Electroporator 2510 (Eppendorf, Germany), and the suspensions were immediately mixed with 1 mL of LB broth and transferred to a microcentrifuge tube for culture at 37 °C with 100–110 rpm shaking for 1 h. About 200 μL of culture was then spread on LB agar plates containing an initial 1.2%, 1.4%, 1.6% and 1.8% (v/v) butanol, respectively. After 16–24 h of 37 °C incubation, the transformants were picked up from these plates and inoculated into 4-mL LB containing 0.7–0.9% butanol for growth evaluation. The best butanol-tolerant mutant was used for subsequent round of shuffling. The pKD46 plasmid was extracted and then transformed to the target strain to provide recombinase system for next round of shuffling (Fig. 1a).

### Butanol tolerance assay

Growth differences among transformants from each round of genome shuffling were preliminarily evaluated in 20-mL test tubes and sealed with a screw cap. The single clones were inoculated into 3-mL LB with 0.5% (v/v) butanol for adaptive growth. These strains and BW25113 were then inoculated into LB for preculture. The overnight seed cultures were transferred to tubes with 4-mL LB containing 0.7 and 0.9% butanol for screening butanol-tolerant mutants in the 1st and 2nd rounds of shuffling, respectively. The cultures were taken each 4 h for determination of the OD_600_ values. The butanol-tolerant strains were picked for further shake-flask evaluation. The cultures of these mutants obtained by each round of shuffling and control strain BW25113 were pre-cultured as aforementioned, and then inoculated to 250-mL screw-cap conical flasks containing 50-mL LB with 0.95% butanol with an initial OD_600_ of 0.1–0.2 for growth evaluation. Butanol tolerance evaluation of BW1847 and BW1857 was performed in the presence of high concentration of butanol (1.6–2.0%). Growth curves of the two strains and control BW25113 were drawn, and their growth improvement was calculated according to the following formula. And the growth improvement of the strain D380 was also evaluated and calculated using this formula.

1 $${\text{growth}}\;{\text{improvement}}\; (\%) = \frac{{{\text{OD}}_{600,\;\text{max} } - {\text{OD}}_{{600,\;{\text{initial}}}} }}{{{\text{OD}}_{{600,\;{\text{initial}}}} }} \times 100$$

### Determination of short-chain alcohol tolerance of BW1847 and BW1857

Seed cultures of BW1847, BW1857 and BW25113 were prepared as aforementioned and then inoculated into 4-mL LB with butanol (0–1.5%), isobutanol (0–1.5%), and ethanol (0–7%), 1-pentanol (0–0.5%), acetic acid (0–0.3%), respectively. Strains were cultured at 37 °C with 200-rpm shaking, and initial OD_600_ was controlled at 0.1. The OD_600_ values at 12 h were determined, and the relative OD_600_ ratios of transformants to control BW25113 cultured under same conditions were calculated to evaluate the butanol tolerance.

### Growth and butanol concentration detection

All growth detection experiments in butanol stress were performed using screw-cap test tubes or conical flasks as aforementioned. Cell density was determined by OD_600_ with a Cary 50 spectrophotometer (Varian Inc.). Butanol concentration in media during culture was detected by high-performance liquid chromatography (HPLC) using Organic Acid Analysis Column (Aminex HPX-87H Ion Exclusion Column, 300 mm × 7.8 mm) at 45 °C and sulphuric acid (4 mM) as mobile phrase at 0.8 mL/min. The butanol concentration at 12 h was subtracted from the initial butanol concentration in the media, and then divided by the OD_600_ value of strain grown at 12 h to obtain the relative butanol content in one cell from each strain, which is used to measure the butanol-efflux ability of cells. All experiments for growth evaluation were performed in triplicates, and statistical differences were analyzed using ANOVA, Single star indicated *p* ≤ 0.05. The heights of the bars and the error bars in figures indicated the means ± standard deviations.

### Evaluation of butanol-tolerance stability of BW1847 and BW1857

Strain BW25113, BW1847 and BW1857 were cultured in LB medium until OD_600_ value reached 4. These strains were then serially transferred ten times (about 50–60 generation) into fresh LB medium with an initial inoculation concentration of 1% (v/v). The serial cultures and the cultures stored in glycerol were all inoculated into 3-mL LB media, and pre-cultured for 12–15 h with 200-rpm shaking. All the pre-cultures were then transferred into 10-mL LB media for the second pre-culture. After 12 h of incubation, the second pre-cultures were inoculated into 250-mL screw-cap conical flask with 50-mL LB containing 0, 0.75, 1.0, 1.25 and 1.5% (v/v) butanol for growth evaluation, respectively. The initial OD_600_ was controlled at 0.2, and the OD_600_ value was determined every 4 h for comparison of growth curves.

### Genome resequencing of mutants and identification of Single Nucleotide Polymorphism (SNP)

Genomic DNA of BW1847 and BW1857 was submitted to Meiji Company (Shanghai) and Novogene (Beijing) for genome resequencing, respectively. Genome sequence of BW25113 (NZ-CP009273.1) was used as a reference sequence for mutation analysis. The sequencing data were deposited in Genebank (SRA accession No. PRJNA397315 and No. PRJNA399799 for BW1847 and BW1857, respectively). The mutated genes were amplified by PCR with 2×Pfu Mastermix (Kangwei Biotech Co. Beijing, China) for sequencing confirmation (Additional file 1: Additional data).

To confirm a deletion of a large gene fragment in genome of BW1857, PCR identification was performed using the genomic DNA of BW1857 as template and primer sets QS-F and QS-R (Additional file 1: Table S1, Additional data), which was located in upstream and downstream of the deleted gene fragment, respectively. The PCR products were then sequencing verified (Sangong Biotech, Shanghai, China).

### Construction of deletion and overexpression strains

Knockout of each mutated gene was performed according to Datsenko et al.’s reports [24] with a minor modification (Additional file 1: Additional data) for functional identification of the mutated genes in BW1847 and BW1857. Overexpression vectors were constructed by ligating the target wild-type gene and its mutated gene into multiple clone sites of pBAD30 vector (as described in Additional file 1: Additional data, Tables S1–S5, Figure S7), yielding the pBAD30-Wgene and the pBAD30-Mgene plasmid, respectively (Table 2). The plasmids were then transferred into strain BW25113 and a target-gene-deleted strain Dgene (Additional file 1: Table S5), and the serial strains were obtained (Table 2), including BW25113 (pBAD30-Wgene), BW25113 (pBAD30-Mgene), BW25113 (pBAD30), Dgene (pBAD30-Wgene), Dgene (pBAD30-Mgene) and Dgene (pBAD30).

### Functional identification of deleted genes in BW1847 and BW1857

Knockout of each gene in the large deleted fragment of BW1857 (Table 1) was performed according to the above description (Additional file 1: Additional data). These mutants (Additional file 1: Table S5) and control BW25113 were inoculated into 3-mL LB liquid media for 12 h pre-culture. The subcultures were then transferred into 25 mL of tubes containing 4-ml LB liquid medium with 0, 0.5%, and 0.75% (v/v) butanol, respectively. The initial OD_600_ was controlled at 0.1, and the OD_600_ were measured at 0 and 12 h to preliminarily evaluate their growth difference. The strains that showed higher cell densities than BW25113 were selected to measure growth curves. These transformants and BW25113 were subcultured as described above, and then transferred into 250-mL conical flasks which contained 50-mL LB liquid medium with 0, 0.5% and 0.75% (v/v) butanol, respectively. The initial OD_600_ was controlled at 0.1, and the OD_600_ value was measured every 1.5 or 2 h to observe the growth difference between transformants and control.

### Site-specific genomic mutation and deletion in *E. coli*

Strains with the large fragment deletion (D123) and partially deleted genes (D1, D2, D3, D12, D13 and D23) were constructed using CRISPR/Cas9 [40] system according to the order of deleted genes on genome (Additional file 1: Tables S5, S7 and S8, Additional data) to detect the combined impact of several deleted genes on 14-kb deletion fragment on growth. Genome-wide site-specific mutant DT385 (mutation of C_1198_T in *acrB* gene) and strain DT900 (AT_686–7_ deletion in rob gene) were constructed using scarless gene modification strategy according to the previous reports [41, 42] with some modification (Additional file 1: Additional data, Table S9, Figure S8).

## Additional file

**Additional file 1.**
