# Supplementary material for: Identification of functional butanol-tolerant genes from Escherichia coli mutants derived from error-prone PCR-based whole-genome shuffling
Source: Biotechnol Biofuels. 2019 Apr 1;12:73. doi: 10.1186/s13068-019-1405-z (PMC6442406; doi:10.1186/s13068-019-1405-z)
Supplement: Supplementary file 1 — Additional file 1. [file 13068_2019_1405_MOESM1_ESM.pdf]

Additional Materials

Identification of functional butanol-tolerant genes from *Escherichia coli* mutants derived from error-prone PCR-based whole genome shuffling

Xueting He[ɨ1,2], Tingli Xue[ɨ1,2], Yuanyuan Ma[1,3,4,5]*, Junyan Zhang[1], Zhiquan Wang[1,2], JiefangHong[1], Lanfeng Hui[6], Jianjun Qiao[4,5], Hao Song[4,5], Minhua Zhang[1,3]

1. Biomass Conversion Laboratory, R&D Center for Petrochemical Technology, Tianjin University, Tianjin 300072, People's Republic of China

2. Department of Biochemical Engineering, School of Chemical Engineering and Technology, Tianjin University, Tianjin 300072, People's Republic of China

3. Frontier Technology Research Institute, Tianjin University, Tianjin 30072

4. Collaborative Innovation Centre of Chemical Science and Engineering (Tianjin), Tianjin, 300072, China

5. Key Laboratory of Systems Bioengineering (Ministry of Education), SynBio Research Platform, Tianjin University, 300072, China

6. Tianjin Key Laboratory of Pulp and Paper, Tianjin University of Science and Technology, Tianjin, China 300457

[ɨ] These authors share co-first authorship and equally contributed to the study.

*** Corresponding author: Yuanyuan Ma**

Address: R&D Center for Petrochemical Technology, Tianjin University, Tianjin，300072, China

Telephone: +86-2287401536;

Fax: +86-2227406119;

E-mail:

For Xueting He: 1134055225@qq.com

For Tingli Xue: 837124173@qq.com

For Yuanyuan Ma: myy@tju.edu.cn

For Junyan Zhang：757833852@qq.com

For Zhiquan Wang：1968996013@qq.com

For Jiefang Hong: hjf@tju.edu.cn

For Lanfeng Hui: huipeak@163.com

For Jianjun Qiao: 674620878@qq.com

For Hao Song: hsong@tju.edu.cn

For Minhua Zhang: mhzhangtju@gmail.com

Additional Materials and Methods

Error-prone PCR genome shuffling

Genomic DNA of *E. coli* BW25113 was extracted and used as the template for error-prone PCR (epPCR) amplification. The epPCR was performed using a ramping procedures according to the previous reports with some modifications (Luhe *et al*. 2011; Ye *et al*. 2013), which included that 10 mer, 12 mer, 14 mer and 15 mer random primers (synthesized by Sangon, Shanghai) were combinatorically used, and 20–50 ng genomic DNA and 5 U of Taq DNA polymerase was added in a final volume of 50 μL. The PCR product amplified by primer pair 12-mer (13.24 μM) and 10-mer (20.06 μM) and that by 15-mer (33.33 μM) had higher DNA concentration than that amplified by other primer sets. Optimal primer combination was used for epPCR amplification, and the PCR products were then concentrated 5–10 times through ethanol-precipitated for electroporation. The transformation was performed as previous described (Huang et al 2018).

In the first round of shuffling, competent cultures were plated on LB plates containing 1.3, 1.4, 1.5 1.6, 1.7, 1.8 and 1.9 % (v/v) butanol for selection of transformants. The transformants were picked up from these plates with 1.3–1.8 % of butanol, and sixty one clones and the initial strain BW25113 were transferred into 25-mL screw-capped tubes containing 3 mL LB for overnight growth and tolerance evaluation in batches. Five percent (v/v) of the overnight cultures of above transformants were then subcultured in 3 mL LB medium containing 0.5 % butanol

for butanol adapted culture. Then, these transformants and BW25113 were precultured in LB media for overnight, and finally transferred into test-tubes containing 4 ml LB media with 0.85 % butanol of for primary growth evaluation. The optical densities at 600 nm (OD_{600}) were measured at 3 and 8 h to evaluate their butanol tolerance. Control strain BW25113 and five strains showing higher cell densities than control strain, were cultured in 250 mL screw-cap conical flask containing 50 mL LB media with 0.95 % butanol for further growth evaluation. Strain BW184 from the plate containing 1.8 % butanol had 33% higher cell density (OD_{600}=0.48) than BW25113 (OD_{600}=0.36) at 7 h, and it was thus used as an initial strain for next round of genome shuffling.

In the second round of shuffling, transformants were screened on plates with 1.8, 1.9, 2.0 and 2.1% butanol. Ninety eight colonies were picked up from plates containing 1.9–2.1% butanol for growth evaluation in test tubes in batches. Eight strains, showing higher cell densities than BW184 and BW25113, were further cultured in conical flask containing LB media with 0.95% butanol for growth evaluation as described aforementioned. The cell densities (OD_{600}=0.64–0.70) of these strains were increased 180–200 % compared with those of control (OD_{600}=0.22). And BW1847(OD_{600}=0.70) and BW1857(OD_{600}=0.68) showed relative higher maximum cell densities than other strains, therefore, the two strains were used for further growth evaluation, resequencing and functional identification of butanol tolerant genes.

Identification of the mutated genes in BW1847 and BW1857

The cassettes of the wildtype and mutated genes were amplified from BW1847 and BW1857 genome using primers listed in Table S2. The reaction system was as followed: 10 ng of genome, 10 pmol each of upstream and downstream primers and 25 μL of 2 × pfu master mix (CW Bio, China) in a final volume of 50 μL. The PCR was performed using a touchdown program: 94°C predenaturation for 5 min; 10 cycles of 1 min denaturation at 94°C, 40 s annealing at 62℃ to 57℃ (-0.5℃/cycle) , and 1–3 min extention at 72°C; followed by 20 cycles of 94°C for 1 min, 57℃ for 40 s, 72℃ for 1–3 min and concluded with a 10 min final extension at 72°C. The extension time was calculated according to the expected product sizes (1 min/kb). Then, a "A" tail was added to the 3' ends of the PCR products at 70℃ for 30 min, and purified by TIANgel Midi Purification Kit (TIANGEN, China). The purified DNA fragments were ligated into a linearized pGEM-Teasy vector (Promega Madison, USA) via TA cloning according to the manufacturer's instructions. Colony PCR was performed for confirmation of insert size. Reaction system was as follows, in a final volume of 20 μL, the reaction mixture contained 0.4 pmol of primers geneF/PUCD1, 0.4 μL Taq DNA polymerase (DingGuo, China), 2 μL 10 × Taq buffer (DingGuo, China) and 0.2 mM dNTP. The reaction program was described as above. The plasmids (Table S3) were extracted using TIANprep Mini Plasmid Kit (TIANGEN, China) and then verified by DNA sequencing (Songon Biotech, Shanghai). The correct clones were designated as shown by Table S3, and the SNPs between mutants and wildtype were also proved by sequencing, as shown in Table 1.

Construction of deletion mutants

Each mutated (Table 1) and deleted gene (Table S1) in BW1847 and BW1857 was knocked out in BW25113 (pKD46) via lambda red recombinant system (Datsenko et al, 2000) for functional complementation experiments. The chloramphenicol resistance cassettes flanked by homologous sequence of target genes were amplified from pKD3 plasmid using serial primers (DF/DR) (Table S4). The target fragments were then electrotransformed into competent BW25113(pKD46）cells, and the transformants were screened on LB plates with 25 μg/mL chloramphenicol. The PCR identification of transformants was performed to detect the deletion of target gene as follows: a freshly isolated colony was suspended in 20 μL PCR reaction mixture containing primer pairs TF/ZY-R, ZY-F/TR and TF/TR (Table S4), respectively. PCR program was described as aforementioned. The primers TF and ZY-R were used to identify the junction between left homologous arm and Cm^R, and primer pair ZY-F/TR were used to amplify the junction between Cm^R and right homologous arm. Primer pair TF/TR was used to verify simultaneous loss of the target gene and gain of a novel DNA fragment (the Cm^R). The deletion mutants obtained were shown in Table S5.

Construction of overexpression vectors

The wild-type and mutated target genes were amplified using serial primer pairs OF/OR (as shown in Table S6), and plasmids obtained by TA cloning (listed in Table S3) as templates. The PCR reaction mixture contained 20 ng of plasmids, 25 μL of 2 × Pfu Master Mix (CWbio, China) and ddH_2O in a final volume of 50 μL. Touchdown PCR was performed using the same conditions described above except that the

annealing temperature decreased from 65°C to 55°C. The pBAD30 plasmid was prepared by the High Pure Maxi Plasmid Kit (TIANGEN, China) according to the manufacturer instruction. The pBAD30 vector and target gene PCR products were digested by same restriction enzyme and purified by TIANgel Midi Purification kit (TIANGEN, China). The target genes were then ligated to the pBAD30 vector through cohesive-end by T4 DNA ligase (Thermo Scientific, USA). And the ligation products were transformed into competent *Escherichia coli* by electroporation, and putative clones were screened on LB agar plates containing 100 μg/mL ampicillin antibiotic. Colonies from each constructs were picked up for PCR confirmation using primer sets pBADIup/OR and OF/pBADIdown (Table S4). The recombinant plasmids were extracted for sequencing verification (Songon Biotech, Shanghai).

The pBAD30 and overexpression plasmids obtained above (Table 2) were transformed into the competent wild *E. coli* BW25113 and the corresponding gene deletion strain (Table S5). The overexpression strains were obtained and named as "name of host stain (plasmid)" (listed in Table 2), and used to growth evaluation. These strains were transferred to 3 mL of LB medium with 100 μg/mL ampicillin antibiotic, 0.5% (v/v) butanol and 0.02% (w/v) L-arabinose for 4–5 h of culture. Then, 15 μL of cultures were added to 15 mL of LB medium as described above without butanol for overnight culture. The cells were harvested by centrifugation (4°C/5000g/2 mins) and resuspended with fresh LB medium. The resuspending culture was inoculated to 50 mL of LB medium contained 100 μg/mL ampicillin antibiotic, 0.02–2% (w/v) L-arabinose and with or without 0.75% (v/v) butanol. The

initial OD_{600} was controlled at 0.1. The cultures were incubated at 37°C with 190 rpm agitation, and the OD_{600} was measured every 1.5 hours. The 0.02% L-arabinose was found to be the optimal induction concentration.

Construction of multiple-gene deletion strains by CRISPR (Clustered regularly interspaced short palindromic repeat sequences)/Cas9 system

Several deletion strains were performed using CRISPR/Cas9 system (Jiang et al, 2015). Inverse PCR was performed using pTargetF as template to introduce the target sequence of N20 (20-bp complementary region) to the upstream of sgRNA in pTargetF plasmid. The N20 sequence was introduced to the primers shown by Table S7. The 50 μL reaction mixture contained 2 ng pTargetF plasmid, 1 μL of 10 μM geneF/Target-R (Table S7), 25 μL of 2 x pfu Master Mix (CW Bio, China). “Touch down” procedure as described above was used except that the annealing temperature decreased from 60°C to 50°C. Then, 0.5 μL DMT enzyme (10 U/μL; GD111, TRANSgene, China) was added into the PCR products, and the mixture were incubated at 37℃ for 1 h to digest methylated template plasmid. The products were purified and transformed into DH5α cells (MCC001, DingGuo, China). After incubation at 37℃ for 1 h, the cultures were plated on LB agar plate with 50 μg/mL spectinomycin. The colonies were confirmed by colony PCR using geneIF/pTarget-IR as primers. The positive pTargetF-geneN20 plasmids (Table S8) were extracted for the subsequent experiment.

The left and right homologous arms of the recombinant fragment were amplified using BW25113 genome as template, geneDLF/geneDLR (left arm) and

geneDRF/geneDRR (right arm) as primer sets, respectively (Table S7). The two homologous arms were fused to a recombinant fragment using overlapping PCR. And the overlap PCR was performed using equal molar ratio of left and right homologous fragments (10–20 ng) as template, and geneDLF/geneDRR as primers. The recombinant fragments were then purified and concentrated to 200 ng/μL for electro-transformation.

The pCas plasmid was transformed into BW25113 competent cells, and the transformants were screened on LB agar plate with 50 μg/mL kanamycin. The positive BW25113 (pCas) colonies were picked up and cultured in LB media with 10 mM L-arabinose (for induction expression of Red recombinase) for preparation of competent cells. When the cells were cultured at 30℃ until the OD_{600} reached 0.375–0.6, the electrically competent cells were prepared. About 50–100 ng of pTargetF-geneN20 plasmid and 500 ng of recombinant fragments were electro-transformed into 40 μL of competent cells. The competent cultures were incubated at 30℃ for 1.5 h and then plated on LB agar plates with 50 μg/mL spectinomycin and kanamycin. The colonies were screened at 30℃ overnight and confirmed by colony PCR using geneIF/geneDRR as primers.

The IPTG was added to media in order to induce the expression of a sgRNA in pCas plasmid, whose expression product could locate on the replicon pMB1 of the pTargetF-geneN20 plasmid (Table S8). The above positive colonies were inoculated into 2 mL LB media with 50 μg/mL kanamycin and 0.5 mM IPTG for 12–15 h cultivation at 30℃ with 200 rpm stirring to eliminate the pTargetF-geneN20 plasmid.

The cultures were then diluted 10^5–10^7-fold with LB media, and 0.1 mL of this dilution was plated on LB agar plate containing 50 μg/mL kanamycin and 0.5 mM IPTG. The clones were further picked up from the agar plates after 12–15 h cultivation, and then point inoculated on LB (with 50 μg/mL kanamycin) agar plates with and without 50 μg/mL spectinomycin, respectively. Clones that could grow on the latter plate but not on the former that were picked up for further elimination of pCas. The positive clones were inoculated into LB media for 12–15 h cultivation at 37℃ with 200 rpm agitation to eliminate temperature-sensitive plasmid pCas. The cultures were then diluted 10^5– 10^7-fold in LB media, and 0.1 mL of the dilution was plated on LB agar plates, and then the single clone was point inoculated on LB agar plates with or without 50 μg/mL kanamycin, respectively. The clones that could grow on LB agar plate and could not grow on that with kanamycin were selected for sequencing confirmation of the edited gene.

Genome-wide mutation of *rob* and *acrB* gene using site-specific mutagenesis

A strain with $AT_{686–7}$ base deletion of *rob* in genome was constructed by site-specific genomic integration (Fig. S8; Zhang et al 2014). The F1 and F4 fragments were PCR-amplified from *E. coli* BW1847 genome with the primers UFrob/URrob and LF/LR, respectively (Table S9). Primers TFrob/T2 and T1/TR were used to amplify F2 and F3 fragments, and the DNA of plasmid pTKS/CS was used as template (Fig. S8; 1st PCR). Amplification was performed in a 50 μL reaction mixture containing 5 ng template, 1 μL of the 10 μM forward and reverse primer, 25 μL of 2 X pfu Master Mix (Kangwei Biotech Co. Beijing, China). After an initial

pre-denaturation at 94℃ for 5 min, 10 cycles of touchdown PCR were performed (denaturation at 94℃ for 30 s, annealing at 65℃ for 30 s with an 0.5℃/cycle decrement until 60℃, and extension at 72℃ for 1 min), followed by 20 cycles of regular PCR (94℃ for 30 s, 30 s at the 60℃, and 72℃ for 1 min) and a final extension step for 10 min at 72℃.

The aforementioned F1 and F2 fragment were fused to the fragment F12 using overlapping PCR. The equal molar ratio of F1 and F2 PCR products (10–20 ng) were used as PCR templates, and UFrob/T2 was used as primers. Fragment F3 and F4 was also fused to the F34 with primer sets T1 and LRrob (Fig. S8; 2st PCR). The final fragment F1234 was amplified through the PCR fusion of the F12 and the F34 fragments using UFrob/LRrob as primers (Fig. S8; 3st PCR). The overlapping PCR was performed using touchdown procedure as described above. The fragment F1234 was then purified and concentrated to 100 ng/μL. Strain BW25113(pTK-RED) was cultured in 50 mL SI-LB (10 g/L tryptone, 5 g/L yeast extract, 10 g/L NaCl, 100 μg/mL spectinomycin and 2 mM isopropyl-β-D-thiogalactopyran oside (IPTG)) medium at 30℃. When its OD_{600} reached 0.375 – 0.6, the competent cells were then prepared. About 5–6 μL of the purified F1234 fragment was then electro-transformed into 40 μL of BW25113(pTK-RED) competent cells. The competent cultures were incubated at 30℃ for 1 h and then plated on SI-LB agar plates with 100 μg/mL tetracycline. The tetracycline resistant mutants were screened and confirmed by colony PCR.

To induce the expression of meganuclease I-SceⅠ and remove the resistance

gene tetA from the genome, the positive colonies were inoculated into 3 mL of SIL -LB media (LB media with 100 μg/mL spectinomycin, 2 mM IPTG and 0.2% w/v L-arabinose) for 12–15 h cultivation. The cultures were then diluted 10^5–10^7-fold in LB media, and 0.1 mL of the dilution was plated on SIL-LB agar plates. Positive clones were picked up from the SIL-LB agar plates, and then point inoculated on SI-LB agar plates with or without 100 μg/mL tetracycline, respectively. Clones, which could grow on SI-LB agar plates without tetracycline but not on that with tetracycline, were selected and confirmed by colony PCR used primers UFrob and LR. The PCR-positive clones were further PCR-amplified using 2 X pfu Master Mix for sequencing verification (Sangong Biotech, Shanghai, China). All primers used were listed in Table S9. The confirmed positive strains were inoculated into LB media and cultured at 42℃ with 200 rpm shaking for 12–15 h in order to eliminate plasmid pTK-RED.

Knockout of the 14-kb DNA fragment

Both BW1857 and BW1847 have a 14-kb DNA fragment deletion. We speculated that the 14-kb deletion can be obtained by the lambda-Red recombination system. Furthermore, the corresponding experiments for knocking out the 14-kb DNA fragment and a control gene (RS18950) were performed using BW25113(pKD46) as a target strain as described in the aforementioned methods. Primers 14kbDF and14kbDR (Table S11) were used to amplify the chloramphenicol resistance cassettes flanked by homologous sequences of the target 14-kb DNA fragment. The target fragments were then electrotransformed into competent BW25113(pKD46）cells,

and the transformants were screened on LB plates with 25 μg/mL chloramphenicol. The PCR identification of transformants was performed to detect the deletion of the 14-kb DNA fragment with primer pairs 14kbTF/ZY-R, ZY-F/395TR and 14kbTF/395TR (Table S11), respectively. Three transformants were picked up for PCR identification using the above three primer sets. The PCR products showed the predicted size bands (Fig. S9), which indicates that the 14-kb DNA fragment was successfully deleted and replaced by a chloromycetin resistance cassette (Fig. S9A). These experiments, thus, demonstrate that the 14-kb deletion in the two mutants may have resulted from homologous recombination. In this experiment, about 40-bp homologous arm sequences could successfully yield crossover recombination, resulting in the knockout of a 14-kb DNA fragment, which means that random PCR products with coincident left (up-stream) and right (down-stream) homologous arm sequences of 14-kb fragments could also yield a crossover homologous recombination. Therefore, the 14-kb deletion could have resulted from crossover recombination via Red recombinase, which is produced from pKD46 plasmid used in the epPCR-based genome shuffling experiment.

Calculation of the mutation rate of BW1847 and BW1857

The spontaneous mutation rate of *E.coli* is reported to be 8.9×10^{-11} per base-pair per generation (Wielgoss et al., 2011), and *E.coli* has a generation period of 15–20 mins. The total time for recovery and screening after electroporation was about 18–20 h (~100 generations), and the final spontaneous mutation rate was 8.9×10^{-9} per base-pair after 100 generation. The final spontaneous mutation rate of the two mutants

(BW1847 and BW1857) was calculated as follows: BW1847 and BW1857 had about 7 and 9 mutations, respectively. The number of mutations (7–9) were divided by 4.6×10^{6} bp of the *E. coli* genome, the final mutation rate obtained, $1.5–2.0\times10^{-6}$ per bp, is about 1000-fold higher than the spontaneous mutation rate (8.9×10^{-9} per base-pair).

Table S1 The deleted genes on the 14-kb deletion fragment of the two mutants

Gene/LC	Function	Deletion strains	Butanol tolerance
ynfH/RS08320	dimethylsulfoxide reductase	D320	-
dmsD/RS08325	twin-argninine leader-binding protein for DmsA and TorA	D325	-
clcB/RS08330	voltage-gated ClC-type chloride channel ClcB	D330	-
ynfK/RS08335	dethiobiotin synthase;	D335	-
dgsA/RS08340	Control and regulate the genes involved in phosphorylation and carbohydrate uptake	D340	decrease 20 % ^{B} and 26 %^{C}
ynfL/RS08345	LysR family transcriptional regulator	D345	-
ynfM/RS08350	MFS transporter; transport the material across the membrane	D350	-
Transposase/RS23865	Transposase	D865	-
Asr/RS08355	acid-shock protein; Required for growth and survival under moderately acidic conditions	D355	-
ydgU/RS08360	stationary phase-induced protein	D360	-
ydgD/RS08365	serine protease; Break peptide bond in large protein molecules	D365	-
mdtI/RS08370	spermidine export protein MdtI	D370	-
mdtJ/RS08375	multidrug transporter subunit MdtJ	D375	decrease 3.5%^{B} and 30%^{C}
TqsA/RS08380	AI-2 transporter TqsA	D380	increase 30%^{C}, 50%^{D} and 22%^{E}
pntB/RS08385	Encode NAD(P) transhydrogenase subunit beta	D8385	-
pntA/RS08390	Encode NAD(P) transhydrogenase subunit alpha		
ydgH/RS08395	DUF1471 family periplasmic protein	D395	-

The butanol tolerance evaluation experiments of the deletion strains of each gene were performed under 0^{A}, 0.5^{B}, 0.75^{C}, 1^{D}, 1.25^{E}% (v/v) butanol stress. The evaluation was performed using the method of the tolerance evaluation of short chain alcohols as described in Method section. The OD_{600} values at 12 h was calculated and shown in the table.

A hyphen “-” indicates that the growth of the target gene deletion mutant hardly differs from that of BW25113

Table S2 Primers for identification of the mutation and deletion in BW1847 and BW1857 genome

Mutated genes	Primer name	Primer sequence
pgsA(RS10005)	005F	5’-CGGGCGAAGGTCAAAAATACCAGTT-3’
	005R	5’-TTCCCGCATTCATCAAGCAATCAGT-3’
yheQ (RS11070)	070F	5’-AATATCGCCAGTGCCGTGGAGTAT-3’
	070R	5’-AGGATATGCCTAATACCGTGGCGTG-3’
hycD (RS14165)	165F	5’-TACTGCCGATGCTCTCTGCTGCTG-3’
	165R	5’-CGACAGCACGTAGTAAACGGCGTAA-3’
aslB (RS19735)	735F	5’-AGTATTTCGGCGAGTAGCGCAGCT-3’
	735R	5’-TTGCACGATCATGTAGGCCGGAT-3’
cdsA (RS00875)	875F	5’-CTGGCCCGATTTCGATGAACAAGA-3’
	875R	5’-AAACGCACGGCATCCCAATCA-3’
acrB (RS02385)	385F	5’-AATGGCACGCTGAAACAAGAGAA-3’
	385R	5’-TCCGTGGTTAATACTGGTTTTCGT-3’
rob (RS22900)	900F	5’-TCTTCTGCATGAGCCAATGGCCCCA-3’
	900R	5’-CTTACCGCAGGGAAGCCGACCGCT-3’
spoT (RS18950)	950F	5’-ACTTCGATACCGCGTTGACCGATT-3’
	950R	5’-CAACGGCATCTGCGGTACGAATAA-3’
musG (RS20660)	660F	5’-TGAGTGCGAATACCGAAGCTCAAG-3’
	660R	5’-AGCTGCAACCTGCAGCTTGACATAG-3’
rplB (RS17195)	195F	5’-CTGAAAAAGCGTCTACTGCGATGGA-3’
	195R	5’-ACCCAGTTTGTGACCAACCATTTCG-3’
infB (RS16425)	425F	5’-CGAAGGGTTGACCGACGAAAAAG-3’
	425R	5’-AGCGTCACATCAGGCAATCCATG-3’
Identification of TA clones	PUCD1	5’-ATACGAGCCGGAAGCATA-3’
Identification of the 14-kb deletion fragment	QS-F	5’-CAGCGTATTGTCCGTGGCATGTTT-3’
	QS-R	5’-GGCATCAAGCCGCCTTATAGGAGT-3’

Table S3 Plasmids used for confirmation of the mutated genes in BW1847 and BW1857

Plasmids	Description	Source or reference
pGEM-Teasy	f1 P_{T7}, Amp^r	Promega
pTW/M070	pGEM-Teasy carries wild(W) and mutated(M) *yehQ*	This study
pTW/M165	pGEM-Teasy carries wild(W) and mutated(M) *hycD*	This study
pTW/M735	pGEM-Teasy carries wild(W) and mutated(M) *aslB*	This study
pTW/M875	pGEM-Teasy carries wild(W) and mutated(M) *cdsA*	This study
pTW/M950	pGEM-Teasy carries wild(W) and mutated(M) *spoT*	This study
pTW/M385	pGEM-Teasy carries wild(W) and mutated(M) *acrB*	This study
pTW/M900	pGEM-Teasy carries wild(W) and mutated(M) *rob*	This study
pTW/M195	pGEM-Teasy carries wild(W) and mutated(M) *rplB*	This study
pTW/M425	pGEM-Teasy carries wild(W) and mutated(M) *infB*	This study
pTW395	pGEM-Teasy carries wild(W) and mutated(M) y*dgH*	This study

Table S4 Primers used for recombination and identification of the deletion mutants

The amplified and verified fragment	Primer name	Primer sequences (homologous arms are underlined)
	ZY-R	5'-TATACCACCGTTGATATATCCC-3'
pgsA-Cm (*RS10005*-Cm)	005DF	5'-TAGGGGTAATCTTACTGACAACAGATAGTTACCCGTCATTATGAGGCTGGAGCTGCTTCG-3'
	005DR	5'-TCACCACTTTTGATCGTTTGCTGAAAATTACGCCGAAACGAATGGGAATTAGCCATGGTCC-3'
	005TF	5'-AAAGTGCCGGGTATTTCGCAAG-3'
yheQ-Cm (*RS11070*-Cm)	070DF	5'-ATTAAACAGTCGATTAAATTGCGCACAAGAGATGGCCCGGCGCAGGCTGGAGCTGCTTC-3'
	070DR	5'-CTATGCCTGACCAATAGCACTCTGTTCCTGATAGCGCCAGCCGATGGGAATTAGCCATGGTCC-3'
	070TF	5'-AATGCTTACATCAGCTTGCGGC-3'
hycD-Cm (*RS14165*-Cm)	165DF	5'-CGGTACTGGTGGTGATTATTGTTTCACGAGGAGCCTGAGAATGAGGCTGGAGCTGCTTC-3'
	165DR	5'-CGCGGCGAGATAATGTTGACCTAATTTTTCTTCAGACATGCTCATGGGAATTAGCCATGGTCC-3'
	165TF	5'-GATGGCGCTGGTTGAACTGG-3'
	165TR	5'-CCCGGAAATGCCTCATTCAG-3'
aslB –Cm (*RS19735*-Cm)	735DF	5'-CCTTAACGTATTGAAGGATGACTTCAGGCAAGGAGCGACCATGAGGCTGGAGCTGCTTC-3'
	735DR	5'-GCCGGATAAGGCGTTTACGCCGCATCCGGCAATCAACCGCAATGGGAATTAGCCATGGTCC-3'
	735TF	5'-TGCCCCACGGTATGATTTCGC-3'
	735TR	5'-CGCTGCAATTTATTGAATTTGCACG-3'
cdsA-Cm *(RS00875*-Cm)	875DF	5'-ACCGAGCCCGGTGATGAAACAGCCTGATGGGGGTCGCTTTTGAGGCTGGAGCTGCTTC-3'
	875DR	5'-AACGAAGCCAAATCCCAGAGAAAACTCAGCATATTACCTTCCGATGGGAATTAGCCATGGTCC-3'
	875TF	5'-CTAATCGAGAGCGTCGTTTCGG-3'
acrB-Cm (*RS02385*-Cm)	385DF	5'-CGAATATGAAAGATGCCATCAGCCGTACGTCGGGCGTGGGTGAAGGCTGGAGCTGCTTC-3'
	385DR	5'-TTCCATGGATGGCAGGCCGTTGTAACGTTCCAGACGCGGCGAAATGGGAATTAGCCATGGTCC-3'
	385TF	5'-GATATCTCCGACTACGTGGCGG-3'
	385TR	5'- GGAATCGACCAGCTCTCGTACA-3'
musG-Cm (*RS20660*-Cm)	660DF	5'-ATGGTGATGAACGACGCGAGCTGGCACCTGGTGCGCAGCGTAAGGCTGGAGCTGCTTC-3'
	660DR	5'-AACCTGGCTGAAGTCCAGCTCTACCGGGGTCGCACGACCGAAGATGGGAATTAGCCATGGTCC-3'
	660TF	5'-CGTCCTCGTTCAGATGGTGATG-3'
rob-Cm (*RS22900*-Cm)	900DF	5'-GTTCCGTATACCGTCAGGATAAACTCCTGCACGCCGGTTCCCAAGGCTGGAGCTGCTTC-3'

(Continued)

The amplified and verified fragment	Primer name	Primer sequences (homologous arms are underlined)
	900DR	5’- AGGCCGGCATTATTCGCGACCTTTTAATCTGGCTGGAAGGTCAATGGGAATTAGCCATGGTCC-3’
	900TF	5’-GACCTTTACGGCGCGTCAGGTT-3’
	900TR	5’- CCGGCATTATTCGCGACCTTTT-3’
spoT-Cm (*RS18950*-Cm)	950DF	5’- GTTTGAATTTATCGAGAGCGTTAAATCCGATCTCTTCCCGGAAGGCTGGAGCTGCTTC-3’
	950DR	5’-GGCGCTGTAGACGCGACCATCTTTCTCTTCCGTATTCAAACATGGGAATTAGCCATGGTCC-3’
	950TF	5’-CGAGAGCGTTAAATCCGATCTC-3’
infB-Cm (*RS16425*-Cm)	425DF	5’-CGGTGACGAAGCGTAATAAACTGTAGCAGGAAGGAACAGCATGAGGCTGGAGCTGCTTC-3’
	425DR	5’-AGCTCCAGAACTTCCGCCTGCAGCAGGATAGCGTCCAGCAGTATGGGAATTAGCCATGGTCC-3’
	425TF	5’-CTGCCCGTAATATTTGCTGGTT-3’
ynfH-Cm (*RS08320*-Cm)	320DF	5’-GATGAAGATGTTTGTATCGGCTGCCGCTACTGCCACATAGGCTGGAGCTGCTTCGAA-3’
	320DR	5’-GTAAAGATAACCAGTGGCCACTCATGCCATCCATTTCCATGGGAATTAGCCATGGTCC-3’
	320TF	5’-AAGATGTTTGTATCGGCTGC-3’
dmsD-Cm (*RS08325*-Cm)	325DF	5’-TGCGGGGCAACCCGCACATTTCAGGATGTTAGGAATGAGGCTGGAGCTGCTTCGAA-3’
	325DR	5’-CGCGTAATCGTCACCATCCGGCAATATTACGGTGATCCTAATGGGAATTAGCCATGGTCC-3’
	325TF	5’-CACATGACAGTAGGAATGGCTA-3’
clcB-Cm (*RS08330*-Cm)	330DF	5’-GCAAAATAGTCGCCCGTGTTTCATTGCCCATTTCTGCTCATGAGGCTGGAGCTGCTTCGAA-3’
	330DR	5’-TTACCGACGCAATTACGCAGGCAATCAATAAACCGGGGAGTAGATGGGAATTAGCCATGGTCC-3’
	330TF	5’-GCAATGGCAATCACAACTGT-3’
ynfK-Cm (*RS08335*-Cm)	335DF	5’-CCTGGTTTATATTTGTGAAGCATAACGGTGGAGTTAGTGATGCTGAGGCTGGAGCTGCTTCG-3’
	335DR	5’-TTCACGCTGTTCGGCGCGCGGCAGATAAGGCAGTTCACCAATATGGGAATTAGCCATGGTCC-3’
	335TF	5’-GCCAGGAAAGCATAACTTAGAC-3’
dgsA-Cm (*RS08340*-Cm)	340DF	5’-TAGCCTACAGATTATTTCGGAGCGCGAAAATATAGGGAGTATGCGGTGAGGCTGGAGCTGCTTCGA-3’
	340DR	5’-TATACATCGCGTCTTTTACCAGTGCAGCGCCTGCCATCGTATGGGAATTAGCCATGGTCC-3’
	340TF	5’-CGTAACTTTCGTATTCATCTGC-3’
ynfL-Cm (*RS08345*-Cm)	345DF	5’-AACTTCGTCATCTGCGTTACTTTGTTGCTGTTGCGGAAGAAGGCTGGAGCTGCTTCG-3’

(Continued)

The amplified and verified fragment	Primer name	Primer sequences (homologous arms are underlined)
	345DR	5’-AAGGGTTCTTTCGCCAGTTCAGCCAGCGTTACATTCGGGTTAATGGGAATTAGCCATGGTCC-3’
	345TF	5’-CAACAGTTGTAGTACGGCTCAC-3’
ynfM-Cm (*RS08350*-Cm)	350DF	5’-CCCTATGTATAAGCCTGATCTACAGGCATATTTAGCAAGGATTTCAAGTGAGGCTGGAGCTGCTTCG-3’
	350DR	5’-GTCCGGACGGGTATTTACCGCAGTCCGGACTTATTTTTCAATGGGAATTAGCCATGGTCC-3’
	350TF	5’-GTAACGCAGATGACGAAGTTC-3’
transposase-Cm (*RS23865*-Cm)	865DF	5’-CGTCTAGCGTCATCAGGATTATAAGTACCCAAATAAACGGATTCAAGGCTGGAGCTGCTTCG-3’
	865DR	5’-GTGACTTAAGGGAAATTTAGCTACCAATAATAGTAGTCTTGATCGGGATGGGAATTAGCCATGGTCC-3’
	865TF	5’-AGCCATCCTAAAATAGACGAAG-3’
asr-Cm (*RS08355*-Cm)	355DF	5’-AGGGATATAGTTATTTCAACGGCCCCGCAGTGGGGTTAAATGAGGCTGGAGCTGCTTCGA-3’
	355DR	5’-AATTTTCAGGCGCGAGGGGGCGCGCCAGCATTACTGTTGAAAACTTAATGGGAATTAGCCATGGTCC-3’
	355TF	5’-GTACATATCGTTACACGCTGAA-3’
ydgU-Cm (*RS08360*-Cm)	360DF	5’-AATTACGGTGCTAAGCGGGTAACGTTTAGCACCGCCTTTAGAGGCTGGAGCTGCTTCGA-3’
	360DR	5’-GTGCGCATAAAATAAGGATGATCAGAATGAACTCAAAGCGATAACGGATGGGAATTAGCCATGGTCC-3’
	360TF	5’-GTTTTCAACAGTAATGCTGGC-3’
ydgD-Cm (*RS08365*-Cm)	365DF	5’-GAGGGTTTGCTTTTAATAATCATAATTACCCACCAGAGTGTGAGGCTGGAGCTGCTTCG-3’
	365DR	5’CCGCGCTGTCGGGCAGCGTTTGAACATTATTTTTGCGACAATGGGAATTAGCCATGGTCC-3’
	365TF	5’-TCCCGTCCCGTCTATAGTATT-3’
mdtI-Cm (*RS08370*-Cm)	370DF	5’-AACCTGAACTGGAGGTGAACCATGGCGCAGTTTGAATGGGTAGGCTGGAGCTGCTTCGA-3’
	370DR	5’-AATAATGTTCAAACGCTGCCCGACAGCGCGGGCAGCGTCTTATGGGAATTAGCCATGGTCC-3’
	370TF	5’-GTTGATAAAATCAGGTACCCGT-3’
mdtJ-Cm (*RS08375*-Cm)	375DF	5’-CGCTGAATTAAGCGAAAATTAAAATAATTCTCTTGCAGGAGAAGGACAATGAGGCTGGAGCTGCTTCGA-3’
	375DR	5’-AAATACCGATACCTTCCCACAGCGCATAAGCTACGCCTAAGGATGGGAATTAGCCATGGTCC-3’
	375TF	5’-CGTACCTCATCCACTATCTTGC-3’
TqsA-Cm (*RS08380*-Cm)	380DF	5’-ACAAGCATGGCAAAGCCGATCATCACGCTCAATGGCCTAAGGCTGGAGCTGCTTCGA-3’

(Continued)

The amplified and verified fragment	Primer name	Primer sequence (homologous arms are underlined)
	380DR	5’-CTGACGGCCTCTGCTGAGGCCGTCACTCTTTATTGAGATCGATGGGAATTAGCCATGGTCC-3’
	380TF	5’-GGATGATAATTGTCCTCGGTAG-3’
pntB-Cm (*RS08385*-Cm)	8385DF	5’-CTCAGCGCATGCTGAAAATGTTCCGCAAAAATTAAGGGGTAACATATGAGGCTGGAGCTGCTTCG-3’
	8385DR	5’-CAATAAAGAGTGACGGCCTCAGCAGAGGCCGTCAGGGTTACATGGGAATTAGCCATGGTCC-3’
	8385TF	5’-GGATTATTGTTGTCGGAGCAC-3’
ydgH-Cm (*RS08395*-Cm)	395DF	5’-ATACCAGTACCTGGTTTGCGCAAGGCGAAGGATTATTTTTATGAGGCTGGAGCTGCTTC-3’
	395DR	5’-GCGAAAGGCGGCATCAAGCCGCCTTATAGGAGTAACTGAATAGATGGGAATTAGCCATGGTCC-3’
	395TF	5’-GCCACGTTTCTCGTTAATAACA-3’
	395TR	5’-GCGGGGAAATTTGTGACTAAAA-3’

Table S5 Knockout strains used in functional study

Strains/ plasmids	Relevant genotype	Source
Strains		
D005	BW25113 △*pgsA*::Cm^r	This study
D070	BW25113 △*yheQ*::Cm^r	This study
D165	BW25113 △*hycD*::Cm^r	This study
D735	BW25113 △*aslB*::Cm^r	This study
D875	BW25113 △*cdsA*::Cm^r	This study
D385	BW25113 △*acrB*::Cm^r	This study
D660	BW25113 △*musG*::Cm^r	This study
D900	BW25113 △*rob*::Cm^r	This study
D950	BW25113 △*spoT*::Cm^r	This study
D425	BW25113 △*infB*::Cm^r	This study
D320	BW25113 △*ynfH*::Cm^r	This study
D325	BW25113 △*dmsD*::Cm^r	This study
D330	BW25113 △*clcB*::Cm^r	This study
D335	BW25113 △*ynfK*::Cm^r	This study
D340	BW25113 △*dgsA*::Cm^r	This study
D345	BW25113 △*ynfL*::Cm^r	This study
D350	BW25113 △*ynfM*::Cm^r	This study
D865	BW25113 △*transposase*::Cm^r	This study
D355	BW25113 △*asr*::Cm^r	This study
D360	BW25113 △*ydgU*::Cm^r	This study
D365	BW25113 △*ydgD*::Cm^r	This study
D370	BW25113 △*mdtI*::Cm^r	This study
D375	BW25113 △*mdtJ*::Cm^r	This study
D380	BW25113 △*tqsA*::Cm^r	This study
D8385	BW25113 △*pntB*::Cm^r	This study
D395	BW25113 △*ydgH*::Cm^r	This study
D1	BW25113 △*ynfH,* △*dmsD,* △*clcB,* △*ynfK*	This study
D2	BW25113 △*dgsA,* △*ynfL,* △*ynfM,* △*transposase,* △*asr,* △*ydgU,* △*ydgD,* △*mdtI,* △*mdtJ*	This study
D3	BW25113 △*tqsA,* △*pntAB,* △*ydgH*	This study
D12	BW25113 △*ynfH,* △*dmsD,* △*clcB,* △*ynfK,* △*dgsA,* △*ynfL,* △*ynfM,* △*transposase,* △*asr,* △*ydgU,* △*ydgD,* △*mdtI,* △*mdtJ*	This study
D13	BW25113 △*ynfH,* △*dmsD,* △*clcB,* △*ynfK,* △*tqsA,* △*pntB,* △*ydgH*	This study
D23	BW25113 △*dgsA,* △*ynfL,* △*ynfM,* △*transposase,* △*asr,* △*ydgU,* △*ydgD,* △*mdtI,* △*mdtJ,* △*tqsA,* △*pntAB,* △*ydgH*	This study
D123	BW25113 with the deleted genes which are also deleted in D1, D2 and D3	This study
Plasmids		
pKD3	Amp^r, FRT, cm^r	Our laboratory
pKD46	Amp^R, ori101(ts), *gam-bet-exo*	Our

laboratory

Table S6 Primers for construction of overexpression plasmids

Primer name	Primer sequences (restriction sites are underlined)
385OF	5’-GTCGC*GAGCTC*ACTTAAACAGGAGCCGTTAAG-3’
385OR	5’-GTGTCCG*AAGCTT*TCAATGATGATCGACAGTATGG-3’
900OF	5’-GGTCGC*GAGCTC*AAAGGATGAGGATATTTTATGGA-3’
900OR	5’-TGTCCG*AAGCTT*TTAACGACGGATCGGAATCAG-3’
165OF	5’-TTCAC*GAATTC*CTGCAA*GAGCTC*TGTTTCACGAGGAGCCTGAGA-3’
165OR	5’-TGTCCG*AAGCTT*AGTAGC*TCTAGA*TCACGCCGCCAGCAAGG-3’
950OF	5’-ATCAT*GAGCTC*TCACAAAGCGGGTCGCC-3’
950OR	5’-CGTCCG*AAGCTT*TTAATTTCGGTTTCGGGTG-3’
070OF	5’-ATTGC*GAGCTC*CGGAGAATCTTCAGTCATG-3’
070OR	5’-GTATCG*GGTACC*TCATCCTTGTTGCTGAAGC-3’
735OF	5’-GTTAC*GAGCTC*AGGCAAGGAGCGACCATG-3’
735OR	5’-GCGTCG*AAGCTT*CCGCCGATTTCTACTTACTCA-3’
875OF	5’-AACAG*GAGCTC*TGGGGGTCGCTTTTGCT-3’
875OR	5’-GCGGCG*AAGCTT*CGTTAAAGCGTCCTGAATACCA-3’
195OF	5’-GTTAC*GAGCTC*GTCGGAGGAGTAATACAATGG-3’
195OR	5’-GTGTCG*AAGCTT*TTATTTGCTACGGCGACG-3’
425OF	5’-GCTTG*GGATCC*TAAACTGTAGCAGGAAGGAAC-3’
425OR	5’-GTGTCCG*AAGCTT*TTAAGCAATGGTACGTTGG-3’
660OF	5’-ATACTC*GAGCTC*CACTGGCCTGAGGTTCTGAG-3’
660OR	5’-TGTCCG*AAGCTT*TTAGGCTTTTTCAACCTGGC-3’
005OF	5’-GGTCGC*GAGCTC*ACAACAGATAGTTACCCGTCATT-3’
005OR	5’-TGTCCG*AAGCTT*TCACTGATCAAGCAAATCTGC-3’
pBADIup	5’-CACACTTTGCTATGCCATAGCA-3’
pBADIdown	5’-ACCGCTTCTGCGTTCTGATT-3’

Table S7 Primers used for construction of deletion strains by genome editing based on CRISPR/Cas9 system

Deletion gene	Primers name	Primer sequences (N20 sequence are underlined)
dmsD	325gF	5’-AGTCCTAGGTATAATACTAGTGCTAATGACGCTTCTGGTAG GTTTTAGAGCTAGAAATAG-3’
	325N20F	5’-GCTAATGACGCTTCTGGTAG-3’
ynfM	350gF	5’-AGTCCTAGGTATAATACTAGTTGAAATACTACTGTTCGCGG GTTTTAGAGCTAGAAATAG-3’
	350N20F	5’-TGAAATACTACTGTTCGCGG-3’
pntB	8385gF	5’-AGTCCTAGGTATAATACTAGTGTGTTTTCCTTGAAGAACAG GTTTTAGAGCTAGAAATAG-3’
	8385N20F	5’-GTGTTTTCCTTGAAGAACAG-3’
	TargetF-R	5’-ACTAGTATTATACCTAGGACTGAG-3’
	pTargetF-IR	5’-ACTGCGGAGCCGTACAAATG-3’
ynfH – *ynfK*	320DLF2	5’-TTGGGTCAATGCGTAGTAGGC-3’
	320DLR2	5’-TAACCGAATCAACCCAGACGGATGAGTGAATAGAC-3’
	335DRF2	5’-GTCTATTCACTCATCCGTCTGGGTTGATTCGGTTA-3’
	335DRR2	5’-CGGTGGAGTTAGTGATGCTGA-3’
	*ynfH*IF	5’-AAGATGTTTGTATCGGCTGC-3’
dgsA – *mdtJ*	340DLF	5’-CCTGCCATCGTGCCCTGGTTAGAA-3’
	340DLR	5’-ATTTATTACCTTGTTTAGCGAACTTGAGCGTCTAACTTC-3’
	375DRF	5’-GAAGTTAGACGCTCAAGTTCGCTAAACAAGGTAATAAAT-3’
	375DRR	5’-GCGGCAAAACGGATACCGCAGA-3’
	*dgsA*IF	5’-ATACAAAGCTTTTAACCCTGCAACAGACGAAT-3’
TqsA – *ydgH*	380IF	5’-ACATAGAATTCTTCGTTATGATGACGGAGCG-3’
	380DLR2	5’-GATTACTTGTTTCTTCTGCCACCAGATAATGAGAAACAC-3’
	395DRF2	5’-GTGTTTCTCATTATCTGGTGGCAGAAGAAACAAGTAATC-3’
	395R3	5’-AAATGGAGTTTGCCACTGAAGCGC-3’
	*mdtJ*IF	5’-AATGTAAGCTTGTTAGCAACGATTTCCAGCAC-3’

Table S8 The plasmids used in genome editing of deletion mutants by CRISPR/Cas9

Plasmids	Description	Source or reference
pCas	*repA101*(Ts) *kana* *Pcas-cas9* P_{araB}-*Red* *lacI*^{q} P_{trc}-sgRNA-*pMB*	Jiang et al, 2015
pTargetF	*pMB1 aadA* sgRNA-*pMB1*	Jiang et al, 2015
pTargetF-325N20	*pMB1 aadA* sgRNA-*dmsD*	This study
pTargetF-350N20	*pMB1 aadA* sgRNA- *ynfM*	This study
pTargetF-385N20	*pMB1 aadA* sgRNA- *pntB*	This study

Table S9 Oligonucleotides used in scarless mutation of *rob* and *acrB* gene in genome

Primer name	Primer sequences (Overlap areas of primers were underlined)
UF rob	5’-TACCTGATGTCAGGTGCTCG-3’
UR rob	5’-CCAGACCTTCAGGTAAACATCACATATTCGCCGCCCTGCAGCATCACCGG-3’
TF rob	5’-CGAATATGTGATGTTTACCTGAAGGTCTGGTAGGGATAACAGGGTAATGT-3’
T2	5’-CTGTAATGCAGGTAAAGCGATC-3’
T1	5’-GTGAAGTGGTTCGGTTGGTT-3’
TR rob	5’-CCAGACCTTCAGGTAAACATCACATATTCGATTACCCTGTTATCCCTACTAA-3’
LF rob	5’-CGAATATGTGATGTTTACCTGAAGGTCTGGGAACCGGCGTGCAGGAGTTT-3’
LR rob	5’-GCACAGACAACTTGGTGCAT-3’
F rob	5’-TCTTCTGCATGAGCCAATGGCCCCA-3’
LR	5’-GCGGGATTGGTGGTCGCACA-3’

Table S10 The relative growth improvement of the mutants

Strains	Tolerant gene	Relative growth improvement	
		0.75%	1.25%
BW25113	Wild type	1	1
DT385 /D385	The mutated/ deleted *AcrB*	1.85/2.03	-
DT900/ D900	The mutated/ deleted *rob*	1.74/1.78	-
DT380	The deleted *TqsA*	0.78	1.68

The strains were cultured in LB media containing 0.75 or 1.25 % (v/v) butanol, and the growth improvement was calculated according to the formula (1) as described in Method section. The value of growth improvement of each strain was divided by that of control BW25113, and the ratio value was shown as the relative growth improvement.

Table S11 Primers used in knockout of 14-kb DNA fragment

Primer name	Primer sequences (The underline bases indicate the homologous arm sequence.)
14kbDF	5'-CTGGCTGTTGATGGGCGTCGGGTTTATTGCCTCTGTCAT*AGGCTGGAGCTGCTTCGAA*-3'
14kbDR	5'-TTCCTGCCACTGGCGGGTGATGTGGTAATACTTAGCACCTT*ATGGGAATTAGCCATGGTCC*-3'
ZY-F	5'-GGTATTCACTCCAGAGCGATGA-3'
395TR	5'-GCGGGGAAATTTGTGACTAAAA-3'
14kbTF	5'-ATGACGAAGTGCGATGGTT-3'
ZY-R	5'-TATACCACCGTTGATATATCCC-3'

Underlined bases indicate the homologous arm sequences.

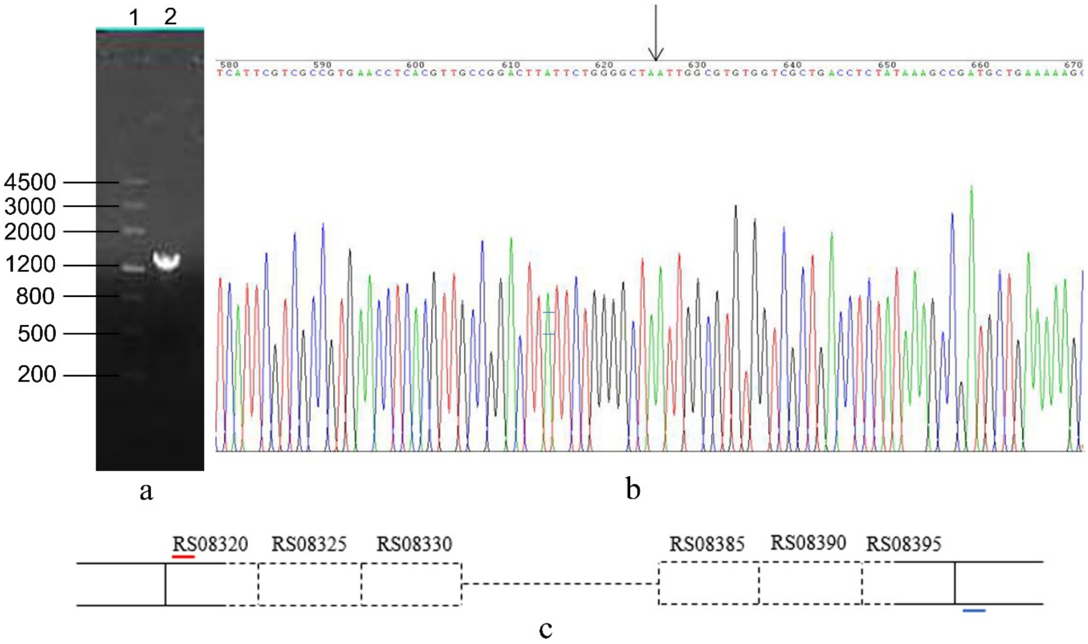

Fig. S1 Molecular identification of the14-kb deletion fragment

a: the electrophoresis map of PCR products using primer pair QS-F and QS-R, which is set in sides of the deleted fragment; b: the sequencing map of the14-kb deletion fragment; c: the deleted genes on the 14-kb deletion fragment, the solid line indicates the gene in genome, and the dotted line represents the deleted gene. The red and blue line indicates the position of the identified primer QS-F and QS-R, respectively.

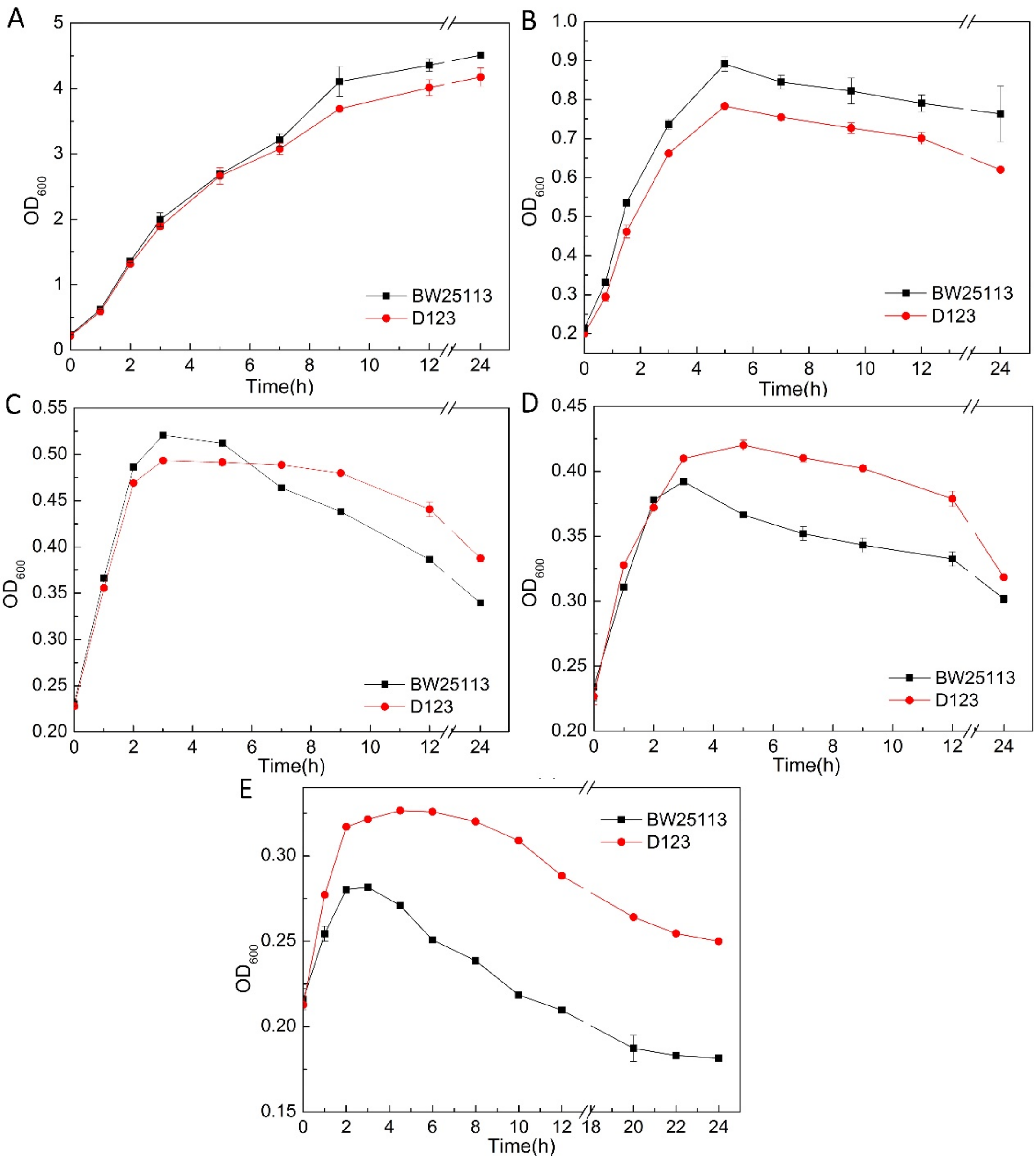

Fig. S2 Growth curves of the strain D123

The strain D123 was cultured in LB medium containing 0 (A), 0.75 (B), 1.0 (C), 1.25 (D) and 1.5 % (E) butanol.

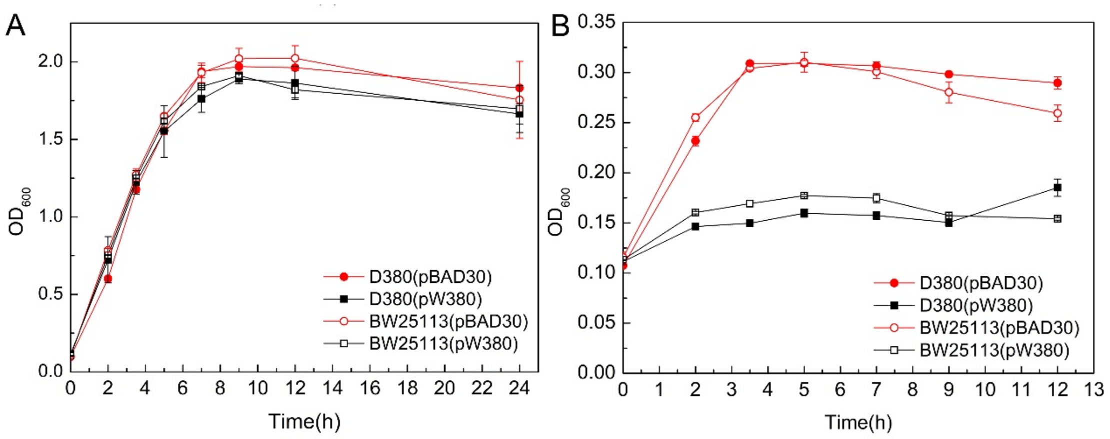

Fig. S3 The growth curves of serial strains for *tqsA* overexpression analysis

The Panel A and B indicates that the strains grown in LB media without and with 0.75% butanol, respectively.

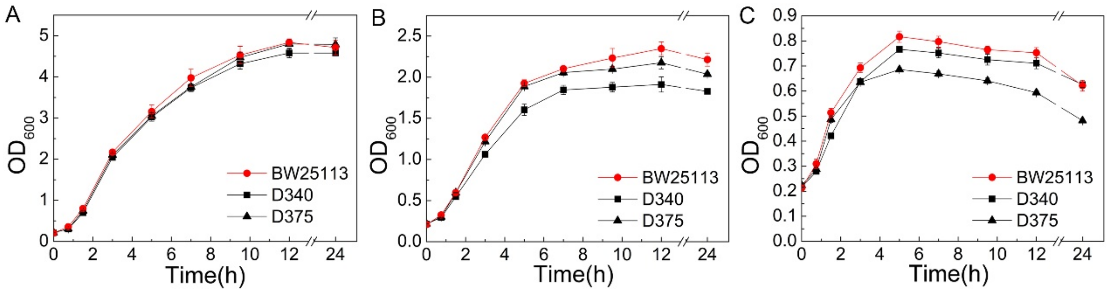

Fig. S4 The growth assay of *dgsA* (RS08340) and *mdtJ* (RS08375) deletion mutants under 0(A)、0.5%(B)、0.75%(C) butanol stress.

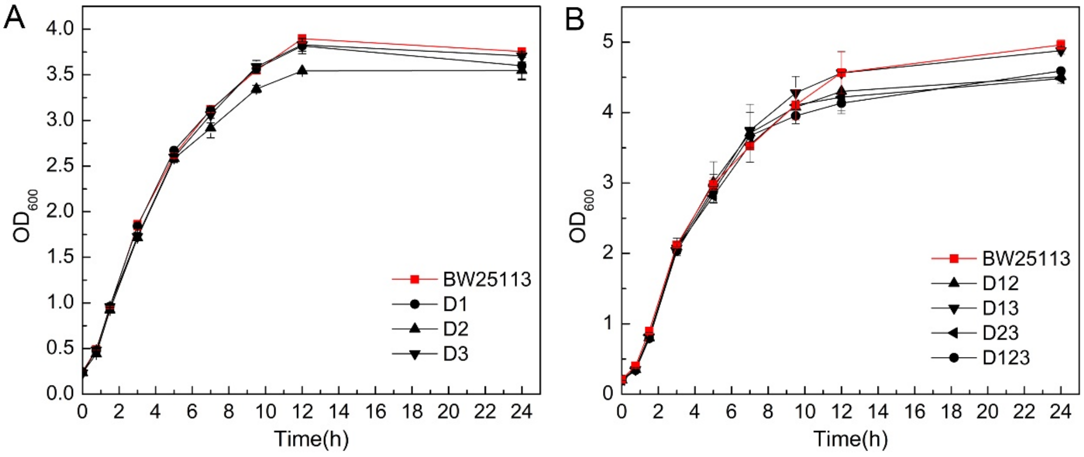

Fig. S5 The growth assay of the combined deletion mutants

The gene type of the strain D1, D2, D3, D12, D13, D23, and D123 are shown in Table S5.

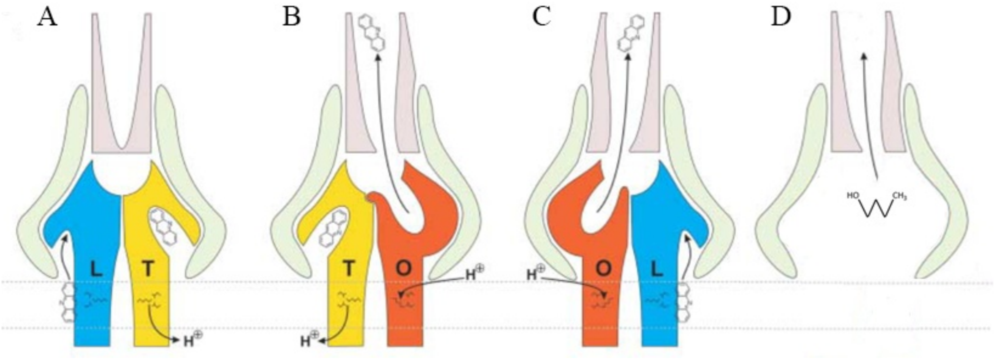

Fig. S6 The putative mechanism of AcrB efflux

The butanol efflux mechanism model was speculated according to the previous report (Seeger et al., 2006). The efflux pump is composed of AcrA, AcrB and AcrC. The AcrA and TolC are indicated in light green and light purple color, respectively. The L, T and O monomer of AcrB is indicated in blue, yellow and red, respectively. The (A), (B) and (C) indicated the substrate transported model showed by Seeger et al.; the mechanism of butanol efflux of DacrB is supposed via Panel (D).

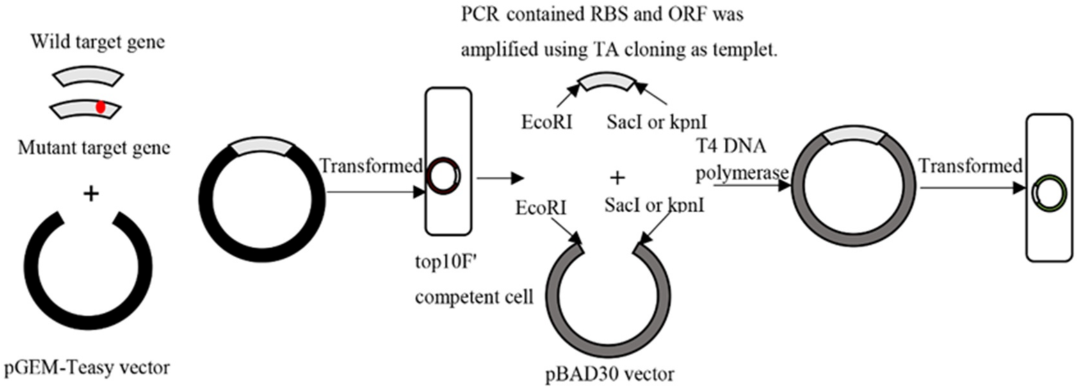

Fig. S7 The strategy map for construction of overexpression strains

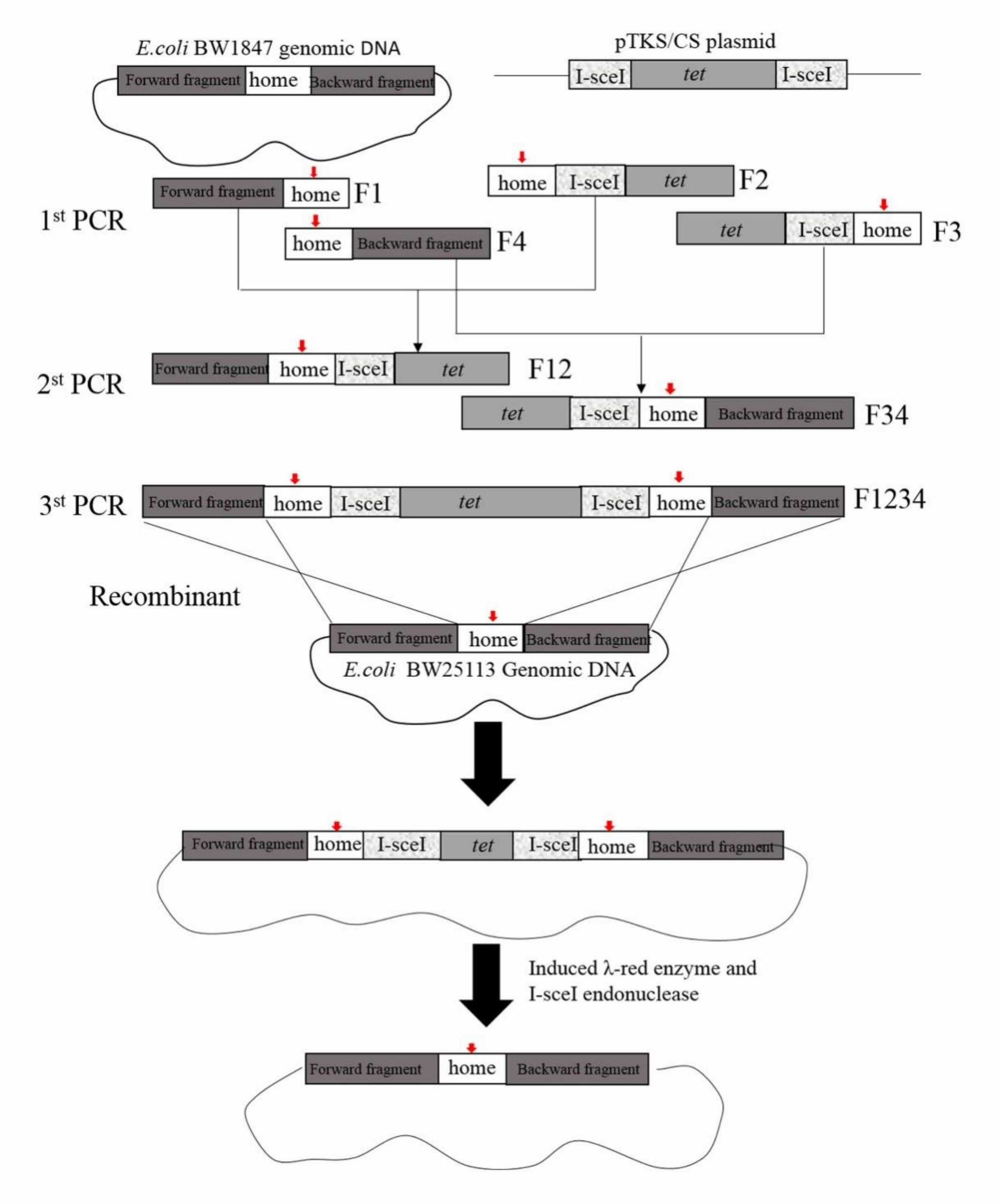

Fig. S8 Strategy for scarless chromosomal point mutations

Genome editing cassette F1234 is obtained by three rounds of PCR, and the recombinant fragment is transferred into the competent cells of target strains. The tetracycline marker gene *tet* is then eliminated by simultaneous induction the expression of the meganuclease I-SceI and the lamda-red recombinase. Mutation sites are indicated by the red arrow.

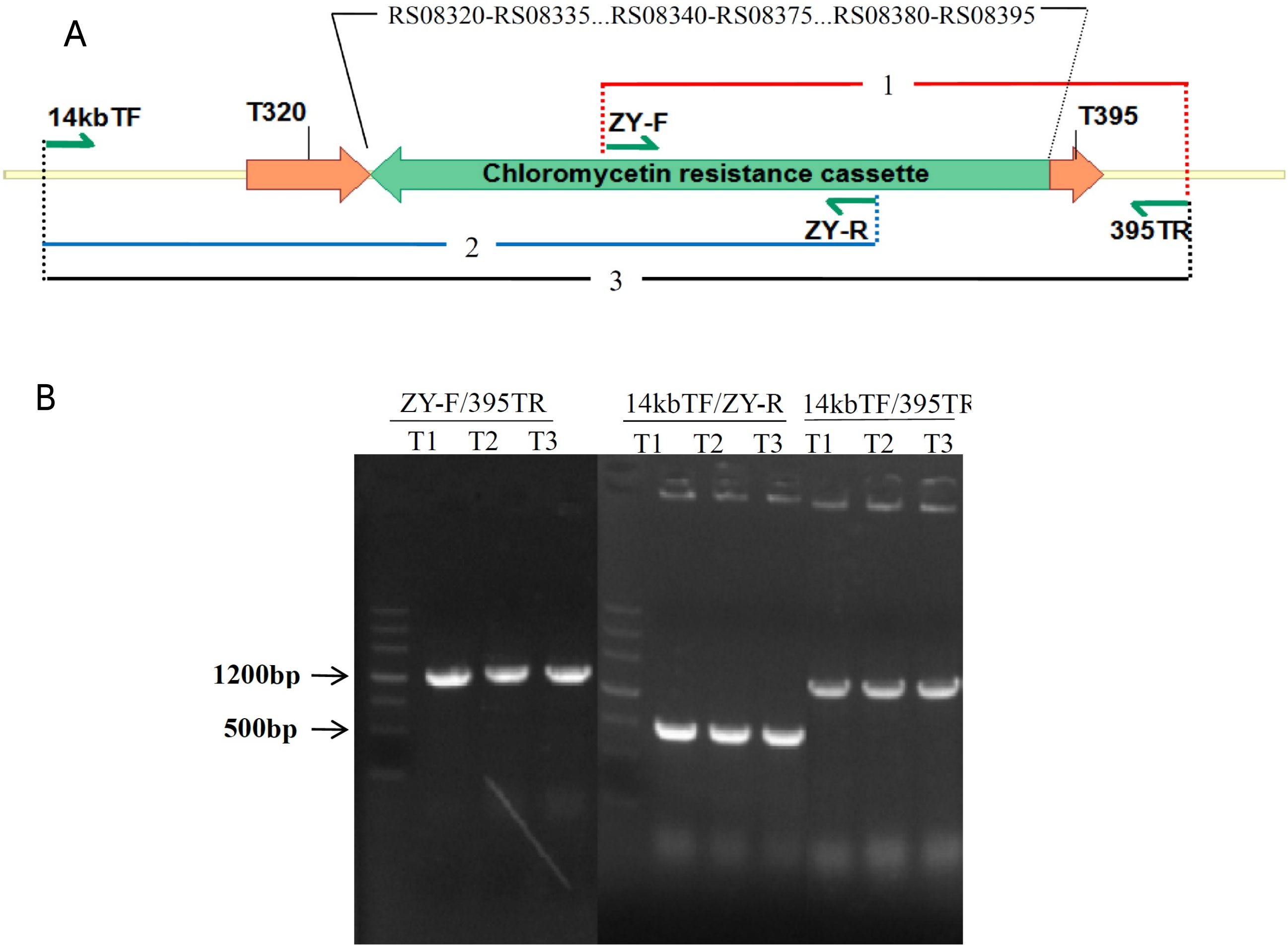

Fig.S9 Primer design and PCR identification of mutants with the 14-kb deletion

Panel A indicates a gene structure shift in transformants with the 14-kb deletion. T320 and T395 indicate the truncated RS08320 and RS08395 genes, respectively. The 14-kb DNA fragment was deleted and replaced by a chloromycetin resistance cassette, using the lambda-Red recombination system. Three primer sets were used to identify transformants with the 14-kb deletion, and the corresponding DNA fragments 1 (red), 2 (blue) and 3 (black) were amplified using the three primer sets. Panel B shows the agarose gel electrophoresis results for PCR identification of transformants. Three clones designated as T1, T2 and T3 were selected for PCR identification using the above primers.

References:

Huang S, Xue T, Wang Z, et al. Furfural-tolerant *Zymomonas mobilis* derived from error-prone PCR-based whole genome shuffling and their tolerant mechanism. Appl Microbiol Biotechnol, 2018, 102(7): 3337 – 3347

Jiang Y, Chen B, Duan C, Sun B, Yang J, Yang S. Multigene editing in the *Escherichia coli* genome via the CRISPR-Cas9 system. Appl Environ Microbiol, 2015, 81(7): 2506 – 2514

Luhe AL, Tan L, Wu J, Zhao H. Increase of ethanol tolerance of *Saccharomyces cerevisiae* by error-prone whole genome amplification. Biotechnol lett, 2011, 33 (5):1077–1011

Seeger MA, Schiefner A, Eicher T, Verrey F, Diederichs K, Pos KM. Structural asymmetry of AcrB trimer suggests a peristaltic pump mechanism. Science, 2006, 313(5791): 1295 – 1298

Wielgoss S, Barrick JE, Tenaillon O, Cruveiller S, Chane-Woon-Ming B, Médigue C, Lenski RE, Schneider D. Mutation rate inferred from synonymous substitutions in a long-term evolution experiment with *Escherichia coli.* G3 (Bethesda). 2011;1(3):183 –186

Zhang Y, Lin Z, Liu Q, Li Y, Wang Z, Ma H, Chen T, Zhao X. Engineering of Serine-Deamination pathway, Entner-Doudoroff pathway and pyruvate dehydrogenase complex to improve poly (3-hydroxybutyrate) production in *Escherichia coli*. Microb Cell Fact, 2014, 13: 172
